# Clonidine used as a perineural adjuvant to ropivacaine, does not prolong the duration of sensory block when controlling for systemic effects: A paired, blinded, randomized trial in healthy volunteers

**DOI:** 10.1371/journal.pone.0181351

**Published:** 2017-09-07

**Authors:** Jakob Hessel Andersen, Pia Jaeger, Tobias Laier Sonne, Jørgen Berg Dahl, Ole Mathiesen, Ulrik Grevstad

**Affiliations:** 1 Department of Anaesthesiology, Zealand University Hospital, Køge, Denmark; 2 Department of Anaesthesiology, Centre of Head and Orthopedics, Rigshospitalet, Copenhagen East, Denmark; 3 Department of Anaesthesiology, Bispebjerg Hospital, Copenhagen NV, Denmark; 4 Department of Anaesthesiology, Gentofte Hospital, Hellerup, Denmark; Université catholique de Louvain, BELGIUM

## Abstract

**Background:**

Clonidine used as an adjuvant to ropivacaine have been shown to prolong the duration of peripheral nerve blocks. The mechanism of action remains unclear. We hypothesized, that clonidine used as an adjuvant to ropivacaine extends the duration of an adductor canal block (ACB) by a peripheral mechanism, compared to ropivacaine alone when controlling for systemic effects.

**Methods:**

We conducted a paired, blinded, randomized trial in healthy volunteers. Participants received bilateral ACBs containing 20 ml ropivacaine 0.5% + 1 ml clonidine 150μg/ml in one leg and 20 ml ropivacaine 0.5% + 1 ml saline in the other leg. The primary outcome measure was duration of sensory block assessed by temperature sensation (alcohol swab). Secondary outcome measures were duration of sensory block assessed by: pinprick, maximum pain during tonic heat stimulation, warmth detection threshold and heat pain detection threshold.

**Results:**

We enrolled 21 volunteers and all completed the trial. There was no difference in duration of sensory block assessed with an alcohol swab: Mean duration in the leg receiving ropivacaine + clonidine was 19.4h (SD 2.7) compared to 19.3h (SD 2.4) in the leg receiving ropivacaine + placebo with a mean difference of 0.1h (95% CI: -1.0 to 1.3), P = 0.83. No differences in block duration were detected when assessed by: Pinprick, mean difference 0.0 h (95% CI: -1.3 to 1.3), maximum pain during tonic heat stimulation, mean difference -0.7 h (95% CI: -2.1 to 0.8), warmth detection threshold, mean difference -0.1 h (95% CI: -1.8 to 1.6) or heat pain detection threshold, mean difference -0.2 h (95% CI: -1.7 to 1.4).

**Conclusions:**

Administering clonidine perineurally as an adjuvant to ropivacaine in an ACB did not prolong the duration of sensory block in a setup controlling for systemic effects of clonidine.

## Introduction

The efficacy of clonidine used as an adjuvant to ropivacaine remains controversial. Some trials report a prolongation in the duration of nerve block [1–3], whereas others do not [4–6]. In-vitro animal studies have demonstrated a perineural effect of block prolongation, mediated through a direct inhibition of the I_h_ current in C-fibers [7], but clonidine also possess systemic analgesic properties.

Peripheral nerve blocks are frequently used for surgical procedures, either as the sole anesthetic, or as part of a multimodal pain management to reduce opioid related side effects [8].

One of the shortcomings of single injection nerve blocks is the relatively limited duration of action. There are several ways of extending the duration of nerve blocks: Continuous peripheral nerve blocks [9, 10], the use of long acting local anesthetics or the addition of different adjuvants [11–13].

A number of studies have shown an increase in postoperative sensory block duration [1–3, 14] and improved postoperative analgesia [13], when adding clonidine to ropivacaine. However, these studies did not control for systemic effects, and whether the difference in duration is caused by local effects, or by a systemic uptake of the peripherally administered clonidine remains unknown. The only clinical study including a systemic control group, added clonidine to ropivacaine in a sciatic-femoral nerve block. This study showed no difference in block duration between the placebo, systemic or perineural groups [6] suggesting no clinically relevant effect of clonidine.

To achieve control of a possible systemic effect of clonidine, we developed a model based on simultaneous bilateral Adductor Canal Blocks (ACBs) in volunteers with 20 ml ropivacaine 0.5% + 1 ml clonidine 150μg/ml in one leg and 20 ml ropivacaine 0.5% and saline in the other leg. As the perineurally administered clonidine on one side was absorbed and redistributed systemically, it would equally affect both nerve blocks in each participant. A longer duration of the nerve block receiving clonidine perineurally would therefore entirely be attributed to a local effect of clonidine on the saphenous nerve.

The aim of the present study was to test the hypothesis that perineural injection of clonidine, used as an adjuvant to ropivacaine, prolongs the duration of an ACB by a peripheral, local mechanism when controlling for a possible systemic effect.

## Materials and methods

**Fig 1:**
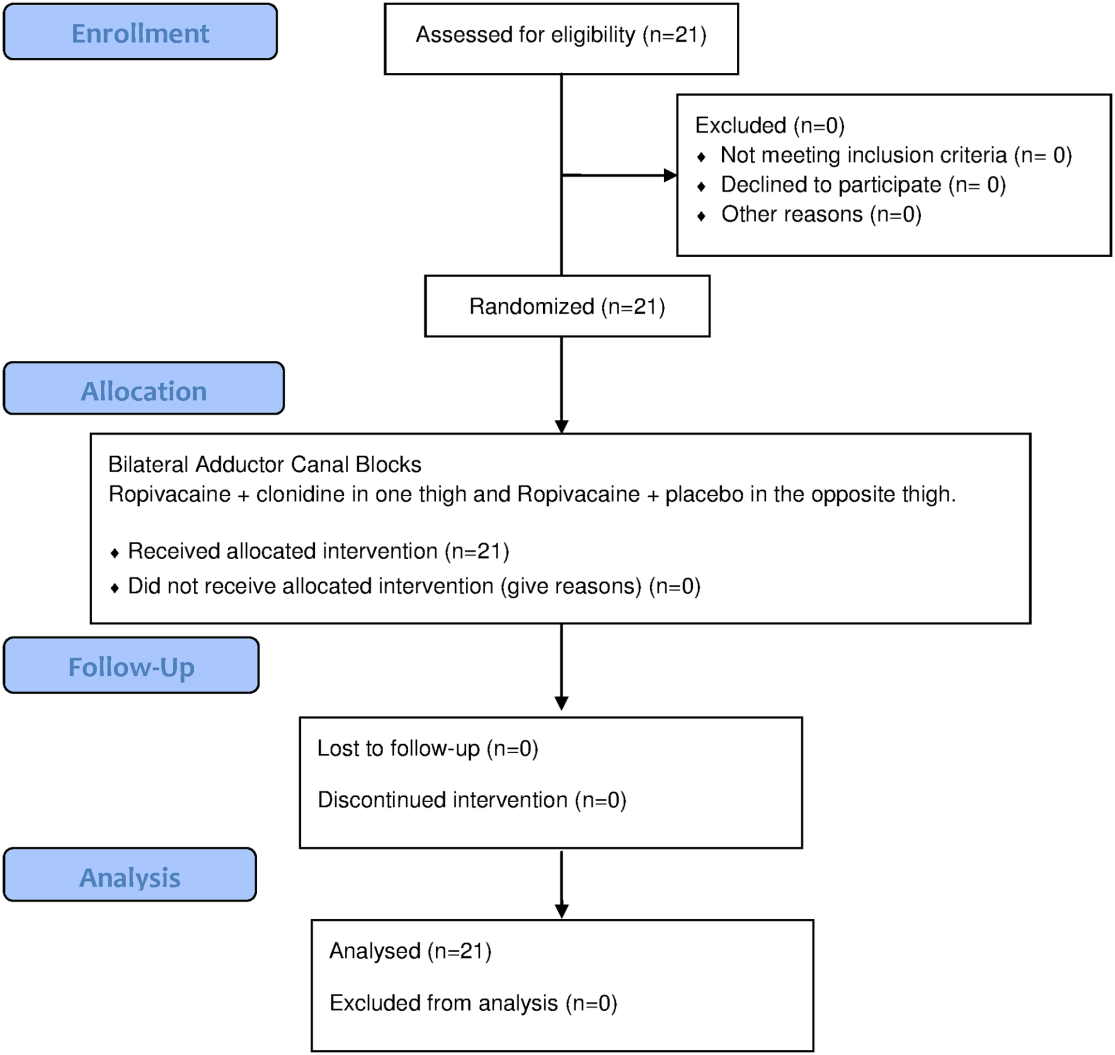
Consolidated Standards of Reporting Trials (CONSORT) flow diagram showing patient progress through the study.

The Regional Ethics Committee (Region Sjællands Videnskabsetiske kommité VEK SJ-437), the Danish Medicines Agency (EudraCT 2014-005640-18) (United States Food and Drug Administration equivalent), and the data protection agency approved the study. Prior to enrollment of the first patient on 05/12/2015, the study was registered at www.clinicaltrials.gov (NCT02444559) by the principal investigator Jakob Hessel Andersen. Signed informed consent was obtained from all subjects. The study design and description are in accordance with the Consolidated Standards of Reporting Clinical Trials (CONSORT) (See CONSORT diagram Fig 1) [15]. The study was monitored by the Copenhagen Good Clinical Practice Unit and conducted according to the Declaration of Helsinki. Clonidine is not approved by the United States Food and Drug Administration or by the European Medicines Agency for perineural use, but is a widely used and accepted adjuvant for perineural administration [13, 16]. In this blinded, randomized paired trial, we planned to include 21 healthy volunteers. Eligible subjects were male, ASA physical status I, age >18 years. Exclusion criteria were: Inability to cooperate, inability to read and speak Danish, known allergies to any of the involved drugs, weekly alcohol consumption of more than 21 units, medical abuse, consumption of opioids within 4 weeks or any analgesics within 48 hours, neuromuscular defects, former surgery or trauma to the lower extremities. Upon screening, the principal investigator included the participants consecutively. The study was conducted in the Perioperative Unit, Department of Anesthesiology, Zealand University Hospital, Køge, in the period May to June 2015. Full protocol is available at www.protocols.io dx.doi.org/10.17504/protocols.io.hsnb6de

### Sensory assessments

The following baseline sensory values were assessed bilaterally in the saphenous nerve distribution on the anteromedial aspect of the lower leg, halfway between the knee and ankle, prior to block performance: The ability to discriminate temperature with an alcohol swab, mechanical discrimination ability with pinprick and three quantitative sensory tests using the Modular Sensory Analyzer Thermal Stimulator (2,5 cm^2^, Thermotest, Somedic A/B, Hörby, Sweden):

*Pain during Tonic Heat Stimulation*: The computer-controlled thermode was heated to 45°C for 30 seconds and the participants were asked to rate their pain (Visual analogue scale score (VAS) 0–100 mm) using a 100 mm horizontal line anchored by no pain at one end and severe pain at the other end. Pain scores were considered normalized when VAS became < 10 mm lower than pre-block values.

*Warmth Detection Threshold* represents the lowest temperature that was perceived as warm. The thermode was heated by 1°C/second from 32°C to a maximum of 52°C. The participants pushed a button when the thermode felt warm and the corresponding temperature was recorded. The test was considered normalized when detection thresholds were less than baseline values + 2°C.

*Heat Pain Detection Threshold* represents the lowest temperature that was perceived as painful. The thermode was heated by 1°C/second from 32°C to a maximum of 52°C. The participants pushed a button when the thermode felt painful, and the corresponding temperature was recorded. The test was considered normalized when detection thresholds was less than baseline valuee + 2°C.

The latter two tests were repeated four times with 6–10 seconds between each measurement and an average was calculated.

After block performance, all sensory tests were repeated every hour post block in the above-described order, until they returned to baseline values. In the period 4 to 10 hours post block (approximately midnight to 6AM) the participants were allowed to sleep and no sensory tests were done. We considered this reasonable, as none of the subjects with an ACB in a previous, similar study had a duration of less than 14 hours. When VAS scores became > 0 during tonic heat stimulation the temperature discrimination test (alcohol swab) was repeated every 30 minutes. Rebound pain, defined as pain during tonic heat stimulation, was measured one hour after normalization of temperature sensation. The principal investigator or the sub investigator did all assessments.

### Block performance

We monitored all participants with continuous ECG, blood pressure and peripheral oxygen saturation every 15 minutes for the first four hours, and thereafter every hour. Sedation was scored hourly according to a verbal Likert scale 0–3 (0 = no sedation, 1 = light sedation, 2 = moderate sedation, 3 = severe sedation). We inserted an intravenous line prior to block performance. Each participant received an ultrasound-guided (Sparq Ultrasound System, Philips Healthcare, Amsterdam, Netherlands) ACB, first in the right leg and then immediately hereafter in the left leg. At the mid-thigh level, the femoral artery was visualized in the short axis below the sartorius muscle. Using an in-plane technique with a 22G x 80 mm needle (Pajunk SonoPlex Stim cannula, Gelsingen, Germany) the sartorius muscle was pierced and study medication was injected slowly anterolateral to the femoral artery surrounding the saphenous nerve in the adductor canal in 5 ml increments preceded by aspiration as described previously[17]. The “adductor canal approach” was chosen since this technique results in a high success rate when blocking the saphenous nerve.[18]

### Study medication

Each participant received bilateral ACBs with 20 ml of ropivacaine 0.5% mixed with 1 ml clonidine 150μg/ml in one leg and 20 ml of ropivacaine 0.5% with 1 ml isotonic saline in the other leg according to randomization in a blinded fashion. Investigators, participants and all other personnel were unaware of the allocation. Skanderborg Pharmacy, Denmark prepared the computer-generated randomization list as well as one box containing study medication for each participant. The boxes were sequentially numbered according to the randomization list. Each box contained two identical ampoules, one containing 1 ml of clonidine 150μg/ml, and the other 1 ml of isotonic saline. According to the randomization list, these ampules were marked left and right leg. The study medication was drawn in syringes marked ‘left’ and ‘right’ leg by the principal investigator and checked by the sub investigator. Blinding was maintained until completion of data analysis.

### Outcome measures

The primary outcome measure was duration of sensory block assessed by temperature sensation (alcohol swab).

Secondary outcome measures were duration of sensory block assessed by: Pinprick, maximum pain during tonic heat stimulation, warmth detection threshold, heat pain detection threshold and difference in rebound pain compared to baseline pain during tonic heat stimulation.

### Statistical methodology

In a previous study by our group using 20 ml of ropivacaine 0.5%, we found a mean duration of the ACB of 22 hours with a standard deviation of four hours [19]. Assuming a correlation of 0.5 between duration for intervention leg and duration for control leg and setting the minimal relevant difference to four hours, α = 0.05 and β = 0.10 a sample size of 18 volunteers would be required. Twenty-one volunteers were included to compensate for possible dropouts.

Statistical analysis was performed using the IBM SPSS Statistics (version 23.0 for Macintosh; Armonk, NY. USA).

According to visual inspection of histograms, normal Q-Q and box plots, Shapiro-Wilks test and test for skewness and kurtosis, all paired differences were approximately normally distributed besides rebound pain. Data are presented as means with 95% confidence intervals. Comparisons are made using paired T-test on all parameters, besides rebound pain which we compare using Wilcoxon signed rank test.

Block failure was defined as a preserved ability to sense cold using an alcohol swab 2 hours post block. A partial block was defined as the inability to sensate temperature when stimulated with an alcohol swab, but VAS > 0 during tonic heat stimulation, at two hours post block.

Sedation and hemodynamic data are only presented descriptively, since all volunteers received clonidine. For individual hemodynamic data see S2 Dataset. Hemodynamic data.

## Results

In the period May-June 2015 twenty-one healthy volunteers provided written consent and were enrolled in the study. No partial blocks or block failures were observed. No participants were excluded and all completed the follow-up period. Participants demographics were (mean (SD)): Age: 23 (2) years, height: 186 (6) cm, and weight: 83 (13) kg with a Body Mass Index of 24 (kg/m^2^). All participants received the intended treatment and could be analysed for every outcome. The trial ended according to plan by the inclusion of the 21. participant. For complete dataset see S1 Dataset. Individual data.

**Fig 2:**
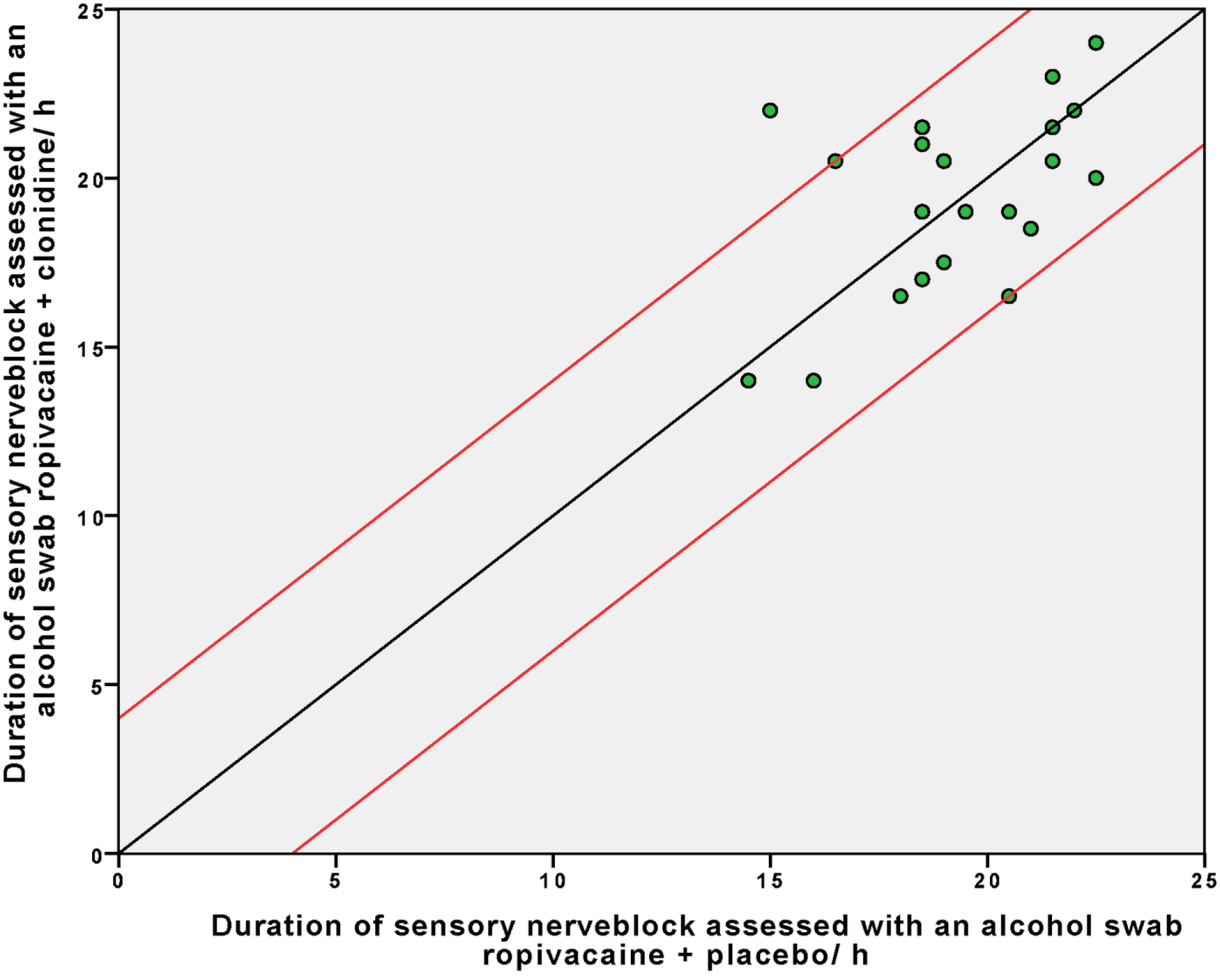
Difference in duration of sensory block between clonidine and placebo assessed using an alcohol swab. Each dot represents the sensory duration of the two blocks from one volunteer, with the ropivacaine + placebo block along the X-axis and ropivacaine + clonidine block along the Y- axis. The black reference line indicates no difference in duration of sensory block between the block receiving clonidine and placebo. Dots above the reference line indicate an advantage of adding clonidine perineurally, dots below indicate an advantage of placebo. The red lines indicate the a priori defined minimal relevant difference of four hours.

There was no difference in duration of sensory block between groups (Fig 2) Mean duration of sensory block was 19.4 h (SD 2.7) in the leg receiving 20 ml ropivacaine 0.5% + clonidine 150 μg compared to 19.3 h (SD 2.4) in the leg receiving 20 ml ropivacaine 0.5% alone, mean difference 0.1 h (95% CI -1.0 to 1.3), P = 0.83. (Table 1). There were no differences among block duration measured by pinprick, tonic heat stimulation, warmth detection threshold or heat pain detection threshold (Table 1). Rebound pain scores, measured one hour after resolution of sensory nerve block, were lower than the baseline scores in both groups (Δ VAS -7 and Δ VAS -6, ropivacaine + placebo and ropivacaine + clonidine, respectively). There was no between-leg difference in rebound pain according to Wilcoxon signed rank test p = 0.97.

**Table 1:** Results.

Sensory test	RopiClonidine Duration (h)	Ropi-Placebo Duration (h)	Mean difference(h)	95% [CI] of thedifference	P
**Temperaturesensation**	19.4	19.3	0.1	-1.0 to 1.3	0.83
**Pinprick**	19.8	19.8	0.0	-1.3 to 1.3	1.00
**Pain during tonic heat stimulation**	19.3	20.0	-0.7	-2.1 to 0.8	0.33
**Warmth Detection Threshold**	20.0	20.1	-0.1	-1.8 to 1.6	0.90
**Heat Pain Detection Threshold**	19.1	19.3	-0.2	-1.7 to 1.4	0.85

Values are presented as time to normalization of the measured sensory tests, according to the methods section, for the block receiving Ropivacaine + Clonidine versus ropivacaine + placebo.

**Fig 3:**
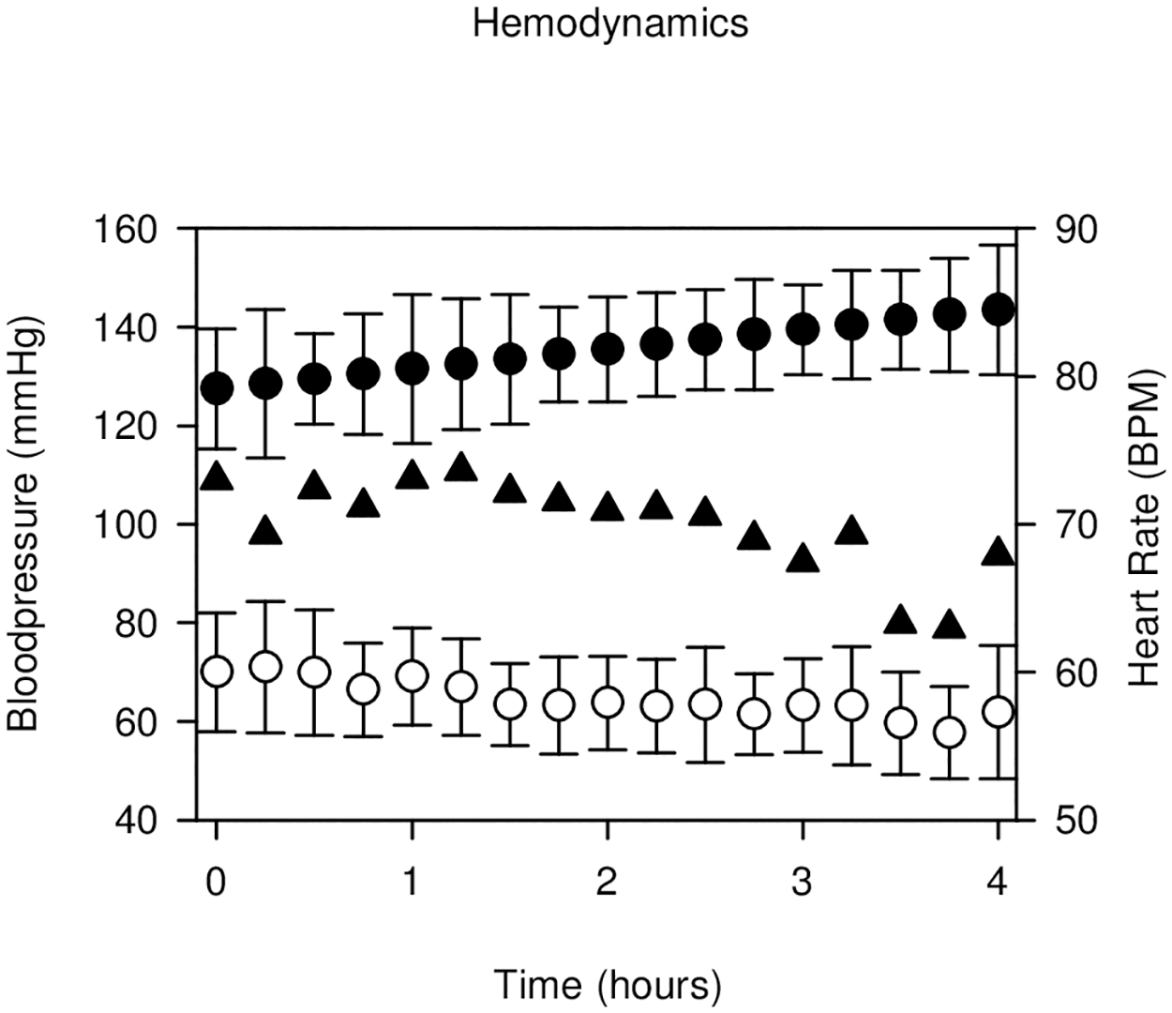
Hemodynamics. During the first four hours of the trial. ○ **Systolic blood pressure** (mean and SD)). ●**Diastolic blood pressure** (mean and SD)). **Heart Rate** (BPM, mean))▲.

Three volunteers experienced bradycardia defined as heart rate < 40 during the trial. All cases occurred between 5 and 10 hours after block performance while the participants were asleep (01–06 am). One participant received a bolus of atropine 0.5 mg IV three times during the night (according to protocol). When he was awakened during these episodes, mean arterial pressure was more than 55 mmHg and he felt no discomfort. The two other cases were self-limiting, hemodynamically stabile and received no treatment, see Fig 3 for hemodynamics and S 2 Dataset. Hemodynamics for individual hemodynamic data. Light sedation occurred in 6 of 21 volunteers during the first 4 hours post block. One participant fell because of reduced strength in one leg.

## Discussion

The results presented in this paper suggest, that there is no advantage of administering clonidine perineurally with ropivacaine, when trying to prolong the duration of an ACB.

The main strength of our trial design is that it ensured control of a potential systemic effect of clonidine on block duration. The clonidine administered perineurally in one leg was absorbed into the systemic circulation, and due to the bilateral setup, systemic effects of clonidine would equally have affected both blocks. Accordingly, if we had seen a longer duration of the nerve block in the leg receiving clonidine perineurally, it would have been caused by a local effect of clonidine. The main limitation of the present design is that it only allowed us to draw firm conclusions had we found a difference in duration. As there was no difference between the groups, there are four possible interpretations of our result: Clonidine has systemic effects, but no local effect. A larger systemic effect overshadowed the local effect. The effects of perineural and systemic clonidine are the same or lastly, that there is no effect of clonidine at all. We observed equal, relatively long durations of nerve blocks, which could be caused by systemic absorption and redistribution of the perineurally administered clonidine. We may have been able to conclude on systemic effects of clonidine as an adjuvant, had we included a placebo-group receiving no clonidine at all.

Most previous trials have employed a two-group design where one group received a block with ropivacaine and clonidine and the other group only ropivacaine [1–5, 14, 20–25]. This type of design investigates the overall effect of clonidine on block duration, but it does not allow us to conclude on the site of action. Results from these trials are conflicting. Four of the trials found no prolongation of the sensory nerve block supporting that clonidine has no effect at all [4, 5, 24, 25], whereas others found a prolonging effect of adding clonidine to ropivacaine [1–3, 14, 20–23]. This effect could be caused by a peripheral mechanism of clonidine, or the absorption and systemic redistribution of the perineurally administered clonidine exerting its properties systemically.

Five trials have aimed to control for possible systemic effects of clonidine by adding a third group. All patients received active nerve blocks with local anesthetic and either perineural-, systemic- or no clonidine (placebo). Again, results are conflicting [6, 26–29].

Helayel et al. [6] used intramuscular clonidine as a systemic adjuvant to ropivacaine in a sciatic nerve block for foot and ankle surgery, whereas Culebras et al. [26] added a group receiving intramuscular clonidine as an adjuvant to bupivacaine in an interscalene block for rotator cuff repair. Both trials found no difference in duration of analgesia between the perineural, systemic and placebo groups, suggesting that there is no effect of clonidine used as an adjuvant.

In a trial on hip fracture patients, all received psoas compartment block with levobupivacaine and either a perineural or an intravenous administration of clonidine or placebo. Administration of intravenous but not perineural clonidine prolonged postoperative analgesia supporting a systemic mechanism of action [27].

Opposed to this, a trial on hand surgery in axillary nerve block combining mepivacaine/ epinephrine with clonidine found significantly longer anesthesia and analgesia in the perineural group compared to placebo and the subcutaneous groups [29]. Similarly, a trial on foot and ankle surgery in popliteal/saphenous nerve block combining bupivacaine/epinephrine with clonidine found significantly longer analgesia in the perineural group compared to placebo and intramuscular groups [28]. These two trials suggest a perineural advantage of clonidine and thus challenges our results.

The problem concerning the three-group design is that it lacks control of the systemic contribution of clonidine, and therefore does not enable us to conclude on the site of action. Both the perineurally- and the systemically administered clonidine would be absorbed, and exert systemic effects. The contradicting results observed in these trials [6, 26–29] could be explained by the pharmacokinetic profile of clonidine. Perineural administered clonidine may have a different rate of absorption, compared to the subcutaneous or intramuscular administered clonidine and could explain the difference in duration of nerve blocks reported between perineural and systemic groups in these studies.

Whether our findings of a lacking advantage of administering clonidine to ropivacaine peripherally can be generalized to other local anesthetics is unknown. One trial compared the addition of clonidine to three different local anesthetics. Clonidine prolonged the duration of motor block in the bupivacaine and mepivacaine groups but not in the ropivacaine group [30]. Pratap et al. performed a bilateral trial on healthy volunteers resembling ours. They injected 0.5 ml of lidocaine 0.5% with 10 μg clonidine subcutaneously on one forearm and 0.5 ml of lidocaine 0.5% with saline subcutaneously on the opposite forearm. The duration of anesthesia was prolonged from 3.5 hours to at least 6 hours when clonidine was added [31]. They assumed that 10 μg of clonidine would be without systemic effects and concluded that clonidine had a peripheral action in enhancing duration of anesthesia on superficial co-infiltration with lidocaine. However, α_2_-receptors are more numerous at peripheral nerve endings compared to axons which may explain the difference in results [32]. Also, lidocaine (as well as bupivacaine) have vasodilator effects and the α_2_-receptor agonists vasoconstriction properties may delay washout of some local anesthetics [33]. Ropivacaine has intrinsic vasoconstriction properties [34] and the additional vasoconstriction mediated when adding clonidine to ropivacaine may not be beneficial.

The systemic adverse events observed in other studies range from no side effects [3, 5, 14, 30] to mild sedation [20] lower heart rate [2, 20, 35] and lower blood pressure [2, 20], and are consistent with our results. These hemodynamic changes are primarily observed during the first four hours. Side effects seems to increase as higher doses of clonidine are used, and 150μg is currently the recommended dose [13]. The bradycardia observed between four and ten hours post block during nighttime in our study is probably physiologically normal for these young healthy males. The participant that fell during the trial had reduced strength in the quadriceps muscle because of proximal spread of study medication in the adductor canal to trigonum femorale eliciting a femoral nerve block.

Our trial has several other limitations than the already mentioned. Since we conducted the study using an almost exclusively sensory nerve block, we cannot comment on clonidine’s effects on motor block. The generalizability to other nerve blocks and whether clonidine could be efficient as a systemic adjuvant requires additional investigation. Further, as no surgery was performed and no inflammatory response or real postoperative pain was present in this trial, generalization to a clinical setting may be difficult. We used 20 ml ropivacaine, which may have led to an insufficient concentration of perineural clonidine (7.5 μM) in order to demonstrate a peripheral effect.

## Conclusion

Clonidine administered as a perineural adjuvant to ropivacaine in an ACB does not prolong the duration of sensory block in a setup controlling for systemic effects of clonidine.

## Supporting information

S1 Text Protocol original language. (PDF)

S2 Text Protocol English. (PDF)

S1 Dataset Individual data. (XLSX)

S2 Dataset Hemodynamic data. (XLSX)

S1 Table CONSORT 2010 checklist. (PDF)
