# Supplementary material for: Clonidine used as a perineural adjuvant to ropivacaine, does not prolong the duration of sensory block when controlling for systemic effects: A paired, blinded, randomized trial in healthy volunteers
Source: PLoS One. 2017 Sep 7;12(9):e0181351. doi: 10.1371/journal.pone.0181351 (PMC5589088; doi:10.1371/journal.pone.0181351)
Supplement: S1 Text — (PDF) [file pone.0181351.s001.pdf]

PROTOKOL

"Forlænger perineural clonidin varigheden af adduktor kanal blok når der kontrolleres for en eventuel systemisk effekt?
- et randomiseret, blindet, parret studie på raske forsøgspersoner"

<u>Forsøgsansvarlig investigator:</u>
Navn: **Jakob Hessel Andersen**
Titel: **Afdelingslæge**

<u>Subinvestigatorer:</u>
Navn: **Pia Jæger**
Titel: **Læge**

Navn: **Tobias Laier Sonne**
Titel: **Reservelæge**

Navn: **Jørgen B. Dahl**
Titel: **Ledende overlæge**

Navn: **Ulrik Grevstad**
Titel: **Forskningsansvarlig overlæge**

<u>Sponsor:</u>
Navn: **Ole Mathiesen**
Titel: **Forskningsansvarlig overlæge**

Diverse navne og adresser på involverede i studiet.

- **Sponsor**:
  Ole Mathiesen
  Forskningsansvarlig overlæge, PhD
  Anæstesiafdelingen, Køge Sygehus
  Lykkebækvej 1
  4600 Køge
  Tlf.: 42376321/23462485
  E-mail: Olemat@dadlnet.dk

- **Forsøgsansvarlig:**
  Jakob Hessel Andersen
  1. Reservelæge
  Anæstesiafdelingen, Køge Sygehus
  Lykkebækvej 1
  4600 Køge
  Tlf.: 60610666
  Email: Jahea@regionsjaelland.dk.
-
- **Subinvestigator:**
  Pia Jæger
  Læge, PhD
  Anæstesi- og Operationsklinikken, HOC, Rigshospitalet
  Blegdamsvej 9
  2100 København Ø
  Tlf.: 3024 3924
  Email: pia.therese.jaeger@regionh.dk

- **Subinvestigator**
  Tobias Laier Sonne
  Reservelæge
  Anæstesiafdelingen, Køge Sygehus
  Lykkebækvej 1
  4600 Køge
  Email: Tobs@regionsjaelland.dk

- **Subinvestigator:**
  Ulrik Grevstad
  Forskningsansvarlig overlæge
  Anæstesiafdelingen
  Gentofte Hospital
  Kildegårdsvej 68
  2900 Hellerup
  Tlf.: 38673892
  Email: ulrik.grevstad@hotmail.com

- **Subinvestigator**
- Jørgen B. Dahl
- Ledende overlæge, dr.med., MMD
- Anæstesiafdelingen, Bispebjerg Hospital
- Bispebjerg Bakke 23
- 2400 København NV
- Tlf.: 21379772
- E-mail: jbdahl@dadlnet.dk

- **Monitor:**
  Københavns Universitetshospitals GCP-enhed
  Bispebjerg Hospital, Bygning 51, 3.sal
  Bispebjerg Bakke 23
  2400 København NV
  Tlf. 3531 3890
  Fax: 3531 3889

- **Samarbejdsparter:**
  Skanderborg Apotek
  Adelgade 27
  8660 Skanderborg

"Forlænger perineural clonidin varigheden af adduktor kanal blok når der kontrolleres for en eventuel systemisk effekt? - et randomiseret, blindet, parret studie på raske forsøgspersoner"

Dato

Undertegnede sponsor bekræfter hermed til enhver tid at følge protokollens ordlyd og arbejde efter de gældende regler for Good Clinical Practice.

Sponsors navn:

Ole Mathiesen
Forskningsansvarlig overlæge
Anæstesiafdelingen, Køge Sygehus
Lykkebækvej 1
4600 Køge

Underskrift og dato:___________________________

Undertegnede forsøgsansvarlig investigator bekræfter hermed til enhver tid at følge protokollens ordlyd og arbejde efter de gældende regler for Good Clinical Practice.

Forsøgsansvarlig investigator:

Jakob Hessel Andersen
1. reservelæge,
Anæstesiafdelingen, Køge Sygehus
Lykkebækvej 1
4600 Køge

Dato:___________________________

PROTOKOLTITEL 1

DIVERSE NAVNE OG ADRESSER PÅ INVOLVEREDE I STUDIET 2

INTRODUKTION OG RATIONALE 7
BAGGRUND 7
LITTERATURLISTE 8
FORMÅL 11
END POINTS 11
PRIMÆRE END POINT 11
SEKUNDÆRE END POINTS 11

ETISKE OVERVEJELSER 11
OVERVEJELSER I FORBINDELSE MED AFPRØVNINGEN SOM HELHED 12
RISICI, BIVIRKNINGER, ULEMPER MV. 13
INFORMATION AF OG SAMTYKKE FRA FORSØGSDELTAGERE 14
BESKYTTELSE AF DATA FRA FORSØGSDELTAGERE 14

RAMMER FOR STUDIET 14
TIDSPLAN 14
STED FOR UNDERSØGELSENS UDFØRELSE 15
STUDIEDESIGN 15

STUDIESELEKTION 15
INKLUSIONSKRITERIER 15
EKSKLUSIONSKRITERIER 15
ØKONOMISKE FORHOLD 16
FORSØGSDELTAGERES GENNEMFØRELSE OG AFBRYDELSE AF FORSØGET 16
ÅRSAGER TIL FORSØGSDELTAGERNES AFBRYDELSE AF FORSØGET 16
PROCEDURE FOR FORSØGSDELTAGERE, DER AFBRYDER FORSØGET 16

METODOLOGI 17
GENEREL BEHANDLINGSPLAN OG MEDICINDOSERING 17
KLINISKE VURDERINGER 18

MEDICIN OG MEDICINHÅNDTERING 19
UNDERSØGELSESMEDICIN 19
BLINDINGSPROCEDURE, PAKNING OG ETIKERING 20
ANDEN BEHANDLING 21
PROCEDURER VED NØDSTILFÆLDE 21
MEDICINHÅNDTERING 21
MEDICINREGNSKAB 21

BIVIRKNINGER 21
BIVIRKNINGER/UØNSKET HÆNDELSER (AES=ADVERSE EVENTS) 22
RAPPORTERING AF AES OG SAES BIVIRKNINGER 23

STATISTISKE ANALYSER 24
BEREGNING AF ANTAL FORSØGSDELTAGERE 24
DATABEARBEJDNING 24

DATAREGISTRERING, SAMT REGLER FOR KONTROL AF UNDERSØGELSESPROCEDURER 24

CASE REPORT FORMS 25
UDDANNELSE 25

YDERLIGERE KRAV OG GENEREL INFORMATION 25
FORSIKRING 25

INTRODUKTION OG RATIONALE

Baggrund

Perifere nerveblokader er hyppigt brugt som en komponent i en multimodal smertebehandling. Fordelene med nerveblokader er udover smertelindring, at de reducerer morfinforbruget og deraf de morfin relaterede bivirkninger som kvalme, opkastning og sedation. Nerveblokader kan udføres enten som en enkelt injektion single-shot (bolus) eller som en kontinuerlig infusion via perineuralt kateter. Begrænsningen ved single-shot blokader er den relativt korte varighed. Sammenlignet med de kateter baserede blokader er single-shot blokader dog muligvis forbundet med en lavere infektions risiko, metoden anses som simplere, og den kan med fordel benyttes til ambulante patienter(1,2).

For at forlænge virkningen af single-shot blokader, har man forsøgt at tilsætte forskellige adjuvanser til lokalanalgetika med varierende succes. Flere studier har vist at clonidin forlænger varigheden af en single-shot nerveblokade sammenlignet med placebo(3–20), men det er uklart om denne effekt udøves via en systemisk eller lokal virkningsmekanisme(3–5,21–23) Vi ønsker derfor at undersøge om perineural administration af clonidin forlænger varigheden af en nerveblokade med ropivacain når der samtidig kontrolleres for en eventuel systemisk effekt.

Vores gruppe har i et endnu ikke publiceret studie valideret forskellige målemetoder der kan benyttes til at vurdere varigheden af en sensorisk blokade, og fundet at følgende målemetoder kan vurdere varigheden med høj præcision: temperaturdiskriminations test med sprit-vædet gaze, pin-prick, bestemmelse af varme-tærskelværdien (Warmth Detection Threshold, WDT) og varme-smerte-tærskelværdien (Heat Pain Detection Threshold, HPDT), samt smerte under lang varme-stimulering af huden.

Clonidins virkningspunkt vil blive undersøgt med udgangspunkt i et adduktor kanal blok (AKB). Dette er en perifer nerveblokade der har vist sig at være effektiv til postoperativ smertebehandling efter knæalloplastik, og som har den fordel at være en næsten ren sensorisk blokade(24–26). Undersøgelsen vil blive udført som et randomiseret, blindet, parret studie på raske forsøgspersoner. Alle deltagere vil få anlagt to AKB med 20 ml 0,5% ropivacain, ét i hvert ben. I henhold til randomiseringen vil der i det ene blok blive tilsat 1,0 ml clonidin 150 µg/ml og i det andet blok 1,0 ml placebo (isotonisk saltvand).
Der er en dosis respons sammenhæng mellem clonidin og forlængelse af nerveblokade. De fleste studier med et positivt resultat har brugt en dosis på 150µg.

Nerveblokader, som smertestillende behandling er anerkendt og gennemprøvet, og et follow-up studie af patienter der havde fået anlagt adduktor kanal blok viste ingen tegn på nerveskade 3-6 måneder efter operationen(27). De modeller der benyttes i studiet er validerede værktøjer indenfor andre specialer, og det ubehag smertestimuli medfører opleves kun som mildt ubehag. Et tidligere forsøg på isolerede sensoriske neuroner fra rotter viste at clonidin ikke var associeret med øget celledød som adjuvans til ropivacain(28).

Litteraturliste

1. Cuvillon P, Ripart J, Lalourcey L, Veyrat E, L'Hermite J, Boisson C, et al. The continuous femoral nerve block catheter for postoperative analgesia: bacterial colonization, infectious rate and adverse effects. Anesth Analg [Internet]. 2001 Oct [cited 2014 Nov 6];93(4):1045–9. Available from: http://www.ncbi.nlm.nih.gov/pubmed/11574381

2. Liu SS, Salinas F V. Continuous plexus and peripheral nerve blocks for postoperative analgesia. Anesth Analg [Internet]. 2003 Jan [cited 2014 Nov 6];96(1):263–72. Available from: http://www.ncbi.nlm.nih.gov/pubmed/12505964

3. Singelyn FJ, Dangoisse M, Bartholomée S, Gouverneur JM. Adding clonidine to mepivacaine prolongs the duration of anesthesia and analgesia after axillary brachial plexus block. Reg Anesth [Internet]. [cited 2014 Nov 6];17(3):148–50. Available from: http://www.ncbi.nlm.nih.gov/pubmed/1606097

4. Barioni MFG, Lauretti GR, Lauretti-Fo A, Pereira NL. Clonidine as coadjuvant in eye surgery: comparison of peribulbar versus oral administration. J Clin Anesth [Internet]. 2002 Mar [cited 2014 Nov 6];14(2):140–5. Available from: http://www.ncbi.nlm.nih.gov/pubmed/11943529

5. Iskandar H, Benard A, Ruel-Raymond J, Cochard G, Manaud B. The analgesic effect of interscalene block using clonidine as an analgesic for shoulder arthroscopy. Anesth Analg [Internet]. 2003 Jan [cited 2014 Nov 6];96(1):260–2, table of contents. Available from: http://www.ncbi.nlm.nih.gov/pubmed/12505963

6. Eledjam JJ, Deschodt J, Viel EJ, Lubrano JF, Charavel P, d'Athis F, et al. Brachial plexus block with bupivacaine: effects of added alpha-adrenergic agonists: comparison between clonidine and epinephrine. Can J Anaesth [Internet]. 1991 Oct [cited 2014 Nov 6];38(7):870–5. Available from: http://www.ncbi.nlm.nih.gov/pubmed/1742820

7. Singelyn FJ, Gouverneur JM, Robert A. A minimum dose of clonidine added to mepivacaine prolongs the duration of anesthesia and analgesia after axillary brachial plexus block. Anesth Analg [Internet]. 1996 Nov [cited 2014 Nov 6];83(5):1046–50. Available from: http://www.ncbi.nlm.nih.gov/pubmed/8895283

8. Bernard JM, Macaire P. Dose-range effects of clonidine added to lidocaine for brachial plexus block. Anesthesiology [Internet]. 1997 Aug [cited 2014 Nov 6];87(2):277–84. Available from: http://www.ncbi.nlm.nih.gov/pubmed/9286891

9. El Saied AH, Steyn MP, Ansermino JM. Clonidine prolongs the effect of ropivacaine for axillary brachial plexus blockade. Can J Anaesth [Internet]. 2000 Oct [cited 2014 Nov 6];47(10):962–7. Available from: http://www.ncbi.nlm.nih.gov/pubmed/11032270

10. Erlacher W, Schuschnig C, Koinig H, Marhofer P, Melischek M, Mayer N, et al. Clonidine as adjuvant for mepivacaine, ropivacaine and bupivacaine in axillary, perivascular brachial plexus block. Can J Anaesth [Internet]. 2001 Jun [cited 2014 Nov 6];48(6):522–5. Available from: http://www.ncbi.nlm.nih.gov/pubmed/11444444

11. Iskandar H, Guillaume E, Dixmérias F, Binje B, Rakotondriamihary S, Thiebaut R, et al. The enhancement of sensory blockade by clonidine selectively added to mepivacaine after midhumeral block. Anesth Analg [Internet]. 2001 Sep [cited 2014 Nov 6];93(3):771–5. Available from: http://www.ncbi.nlm.nih.gov/pubmed/11524354

12. Adnan T, Elif AA, Ayşe K, Gülnaz A. Clonidine as an adjuvant for lidocaine in axillary brachial plexus block in patients with chronic renal failure. Acta Anaesthesiol Scand [Internet]. 2005 Apr [cited 2014 Nov 6];49(4):563–8. Available from: http://www.ncbi.nlm.nih.gov/pubmed/15777307

13. Iohom G, Machmachi A, Diarra D-P, Khatouf M, Boileau S, Dap F, et al. The effects of clonidine added to mepivacaine for paronychia surgery under axillary brachial plexus block. Anesth Analg [Internet]. 2005 Apr [cited 2014 Nov 6];100(4):1179–83. Available from: http://www.ncbi.nlm.nih.gov/pubmed/15781541

14. Reinhart DJ, Wang W, Stagg KS, Walker KG, Bailey PL, Walker EB, et al. Postoperative analgesia after peripheral nerve block for podiatric surgery: clinical efficacy and chemical stability of lidocaine alone versus lidocaine plus clonidine. Anesth Analg [Internet]. 1996 Oct [cited 2014 Nov 6];83(4):760–5. Available from: http://www.ncbi.nlm.nih.gov/pubmed/8831317

15. Casati A, Magistris L, Fanelli G, Beccaria P, Cappelleri G, Aldegheri G, et al. Small-dose clonidine prolongs postoperative analgesia after sciatic-femoral nerve block with 0.75% ropivacaine for foot surgery. Anesth Analg [Internet]. 2000 Aug [cited 2014 Nov 6];91(2):388–92. Available from: http://www.ncbi.nlm.nih.gov/pubmed/10910854

16. Madan R, Bharti N, Shende D, Khokhar SK, Kaul HL. A dose response study of clonidine with local anesthetic mixture for peribulbar block: a comparison of three doses. Anesth Analg [Internet]. 2001 Dec [cited 2014 Nov 6];93(6):1593–7, table of contents. Available from: http://www.ncbi.nlm.nih.gov/pubmed/11726451

17. Bharti N, Madan R, Kaul HL, Khokhar SK, Mishra S. Effect of addition of clonidine to local anaesthetic mixture for peribulbar block. Anaesth Intensive Care [Internet].

2002 Aug [cited 2014 Nov 6];30(4):438–41. Available from: http://www.ncbi.nlm.nih.gov/pubmed/12180581

18. Naja ZM, Ziade FM, El-Rajab MA, Naccash N, Ayoubi J-M. Guided paravertebral blocks with versus without clonidine for women undergoing breast surgery: a prospective double-blinded randomized study. Anesth Analg [Internet]. 2013 Jul [cited 2014 Nov 6];117(1):252–8. Available from: http://www.ncbi.nlm.nih.gov/pubmed/23632052

19. Yazbeck-Karam VG, Siddik-Sayyid SM, Abi Nader EL, Barakat DE, Karam HS, Cherfane GM, et al. Supplementation of retrobulbar block with clonidine in vitreoretinal surgery: effect on postoperative pain. J Clin Anesth [Internet]. 2011 Aug [cited 2014 Nov 6];23(5):393–7. Available from: http://www.ncbi.nlm.nih.gov/pubmed/21802630

20. YaDeau JT, LaSala VR, Paroli L, Kahn RL, Jules-Elysée KM, Levine DS, et al. Clonidine and analgesic duration after popliteal fossa nerve blockade: randomized, double-blind, placebo-controlled study. Anesth Analg [Internet]. 2008 Jun [cited 2014 Nov 6];106(6):1916–20. Available from: http://www.ncbi.nlm.nih.gov/pubmed/18499632

21. Culebras X, Van Gessel E, Hoffmeyer P, Gamulin Z. Clonidine combined with a long acting local anesthetic does not prolong postoperative analgesia after brachial plexus block but does induce hemodynamic changes. Anesth Analg [Internet]. 2001 Jan [cited 2014 Nov 6];92(1):199–204. Available from: http://www.ncbi.nlm.nih.gov/pubmed/11133627

22. Mannion S, Hayes I, Loughnane F, Murphy DB, Shorten GD. Intravenous but not perineural clonidine prolongs postoperative analgesia after psoas compartment block with 0.5% levobupivacaine for hip fracture surgery. Anesth Analg [Internet]. 2005 Mar [cited 2014 Nov 6];100(3):873–8, table of contents. Available from: http://www.ncbi.nlm.nih.gov/pubmed/15728081

23. Pratap JN, Shankar RK, Goroszeniuk T. Co-injection of clonidine prolongs the anesthetic effect of lidocaine skin infiltration by a peripheral action. Anesth Analg [Internet]. 2007 Apr [cited 2014 Nov 6];104(4):982–3. Available from: http://www.ncbi.nlm.nih.gov/pubmed/17377118

24. Jæger P, Grevstad U, Henningsen MH, Gottschau B, Mathiesen O, Dahl JB. Effect of adductor-canal-blockade on established, severe post-operative pain after total knee arthroplasty: A randomised study. Acta Anaesthesiol Scand. 2012;56:1013–9.

25. Jenstrup MT, Jæger P, Lund J, Fomsgaard JS, Bache S, Mathiesen O, et al. Effects of Adductor-Canal-Blockade on pain and ambulation after total knee arthroplasty: A randomized study. Acta Anaesthesiol Scand. 2012;56:357–64.

26. Jaeger P, Nielsen ZJK, Henningsen MH, Hilsted KL, Mathiesen O, Dahl JB. Adductor canal block versus femoral nerve block and quadriceps strength: a randomized, double-blind, placebo-controlled, crossover study in healthy volunteers. Anesthesiology. 2013;118:409–15.

27. Henningsen MH, Jaeger P, Hilsted KL, Dahl JB. Prevalence of saphenous nerve injury after adductor-canal-blockade in patients receiving total knee arthroplasty. Acta Anaesthesiol Scand [Internet]. 2013;57:112–7. Available from: http://www.ncbi.nlm.nih.gov/pubmed/23074997

28. Williams BA, Hough KA, Tsui BYK, Ibinson JW, Gold MS, Gebhart GF. Neurotoxicity of adjuvants used in perineural anesthesia and analgesia in comparison with ropivacaine. Reg Anesth Pain Med [Internet]. [cited 2014 Oct 27];36(3):225–30. Available from: http://www.pubmedcentral.nih.gov/articlerender.fcgi?artid=3085859&tool=pmcentrez&rendertype=abstract

29. McCartney CJL, Duggan E, Apatu E. Should we add clonidine to local anesthetic for peripheral nerve blockade? A qualitative systematic review of the literature. Reg Anesth Pain Med [Internet]. [cited 2014 Nov 6];32(4):330–8. Available from: http://www.ncbi.nlm.nih.gov/pubmed/17720118

Formål

Vores formål er at undersøge om clonidin tilsat som et adjuvans til ropivacain ved et AKB kan forlænge varigheden af sensorisk blokade via en perifer virkning når der samtidig kontrolleres for en eventuel systemisk effekt. Vores hypotese er at clonidin tilsat som adjuvans til ropivacain forlænger varigheden af et AKB ved perineural injektion sammenlignet med ropivacain i kombination med placebo.

Endpoints

Det primære effektmål

- Forskellen i varighed af sensorisk blokade imellem AKB med ropivacain + clonidin versus AKB med ropivacain + placebo, målt som tiden fra blok-anlæggelsen (fjernelse af nålen) til sprit-vædet gaze igen mærkes som koldt (temperaturdiskrimination).

Sekundære effektmål

- Forskellen i varighed af sensorisk blokade imellem AKB med ropivacain + clonidin versus AKB med ropivacain + placebo, målt som tiden fra blok-anlæggelsen til pin-prick af huden igen udløser et sensorisk stimuli (føles spids).
- Forskellen i varighed af sensorisk blokade imellem AKB med ropivacain + clonidin versus AKB med ropivacain + placebo, målt som tiden fra blok-anlæggelse til smertescore

ved lang varme-stimulering af huden igen returnerer til udgangsværdien (± 10 mm i VAS).

- Forskellen i varighed af sensorisk blokade imellem AKB med ropivacain + clonidin versus AKB med ropivacain + placebo, målt som tiden fra blok-anlæggelse til tærskelværdien for varme-udløst smerte (HPDT) returnerer til udgangsværdien(+2° C, eller derunder).
- Forskellen i varighed af sensorisk blokade imellem AKB med ropivacain + clonidin versus AKB med ropivacain + placebo, målt som tiden fra blok-anlæggelse til tærskelværdien for varme-detektering (WDT) returnerer til udgangsværdien (+2° C, eller derunder).
- Forskellen i maksimum smertescore (VAS) ved lang varme-stimulering af huden (30 sekunder ved 45° C), imellem AKB med ropivacain + clonidin versus AKB med ropivacain + placebo, til tiden 4 timer post-blok.
- Forskellen i maksimum smertescore (VAS) ved lang varme-stimulering af huden (30 sekunder ved 45° C), imellem AKB med ropivacain + clonidin versus AKB med ropivacain + placebo, 1h efter værdien er returneret til udgangsværdien (rebound pain).

ETISKE OVERVEJELSER

Overvejelser i forbindelse med afprøvningen som helhed

Ropivacain er et kendt og indregistreret lægemiddel, og gives indenfor den anbefalede dosering.

Clonidin er kendt og indregistreret til systemisk behandling. Clonidin er ikke indregistreret til perineural administration, om end det i litteraturen er et hyppigt undersøgt præparat ved anlæggelse af nerveblokader. Der findes ingen rapporter af clonidin induceret nerveskade efter nerveblokader tilsat clonidin perineuralt, og et *in vitro* studie på rotter viste at ropivacain og clonidin i kliniske koncentrationer ikke øger risikoen for nerveskade sammenlignet med ropivacain alene. Det derfor vores vurdering, at bivirkninger og risici ved deltagelse i denne undersøgelse er minimale.
De anvendte blokader anlægges ultralydsvejledt og skønnes ikke forbundet med komplikationer. Blokaderne anlægges af én speciallæge i anæstesiologi med stor erfaring indenfor ultralydsvejledte blokader.

De akutte smerteinduktionsmodeller, der benyttes i dette forsøg, er sikre, simple og reproducerbare og gør dem egnet til at undersøge varigheden af nerveblokader.

Der vil i dette forsøg blive brugt raske forsøgsdeltagere frem for patienter, da studiet skal udføres som et parret forsøg, for at undersøge om perineural clonidin har en lokal effekt når det samtidig kontrolleres for en evt. systemisk effekt af clonidin efter perineural indgift.

I dette forsøg ønsker vi at efterprøve hypotesen om at clonidin tilsat som et adjuvans til ropivacain ved en perifer nerveblokade kan forlænge varigheden af blokaden via en lokal virkning, sammenlignet med placebo. Forsøgspersonerne vil ikke direkte drage fordel af forsøget, men vi anser dette for en meget vigtig undersøgelse, idet vi mener at det er vigtigt at få afklaret om clonidin virker lokalt. Dersom det viser sig at perineural tilsætning af clonidin som adjuvans til ropivacain forlænger varigheden af nerveblokaden vil det bidrage

til en forbedret postoperativ smertebehandling, modsat vil uvirksom behandling kunne undgås dersom vi finder at perineural injektion af clonidin i kombination med ropivacain ikke forlænger blokadens varighed.

Undersøgelsen vil blive udført i overensstemmelse med principperne i Helsinki Deklarationen. Protokollen vil blive indsendt til den lokale Videnskabsetiske Komité, Sundhedsstyrelsen samt Datatilsynet til godkendelse. Investigator vil desuden informere den Videnskabsetiske komité, Sundhedsstyrelsen og Datatilsynet om væsentlige eller større ændringer i protokollen. Undersøgelsen vil blive anmeldt på den internationale database www.eudract.ema.europa.eu samt clinicaltrial.gov.

Risici, bivirkninger, ulemper mv.

Som alle procedurer er også anlæggelsen af perifere nerveblokader forbundet med komplikationer, der dog ses ekstremt sjældent (opgøres mellem 0,02-0,4 %).

Komplikationer kan ses i form af:

- Nerveskade. Ca. 99 % af nerveskader er væk efter 1 år.
- Ved korrekt brug har ropivacain ses ikke væsentlige bivirkninger, men ved accidentiel intravaskulær injektion kan der ses forgiftningssymptomer, som spænder fra paræstesier til kramper og kredsløbskollaps. Risikoen for intravasal injektion af lokalanalgetikumet er meget lille og der sikres mod dette ved aspiration inden indgift og efter hver injektion med 5 ml lokalanalgetikum
- Systemiske bivirkninger til clonidin er forbigående og oftest milde. Sedation opleves typisk ikke ubehageligt. Hypotension (MAP <55) kan behandles med indgift af isotonisk natriumchlorid eller medikamentelt med indgift af efedrin 10mg iv. Bradycardi kan forekomme men er sjældent behandlingskrævende. Behandlingen af betydende bardycardi, defineret som HR< 40, består af indgift af atropin 0.5mg iv.
- Der kan forekomme et mindre hæmatom svarende til indstiksstedet. Dette kan medføre let ømhed og forsvinder uden behandling.

De anvendte blokader anlægges ultralydsvejledt, hvilket formentlig reducerer risikoen for komplikationer.

Der er en dosis respons sammenhæng mellem clonidin og forlængelse af nerveblokade. De fleste studier med et positivt resultat har brugt en dosis på 150μg, og her er der samtidig rapporteret et minimum af bivirkninger(29). Clonidin er et hyppigt anvendt præparat i det daglige på sygehus. Bl.a. til behandling af rysteture (shivering) på opvågningsafsnit efter operation og mod abstinenser. Ved doser på 300 μg ses mere betydende sedation og hæmodynamisk instabilitet(29).

Der anlægges desuden en venflon, dette kan medføre en let svie og muligvis et lille hæmatom efter fjernelse af dette.

Et tidligere studie har vist at efter anlæggelse af et adduktor kanal blok vil der kun ses en mindre motorisk blokade der ikke vil genere forsøgspersonens mobilitet(26). Forsøgspersonen vil blive observeret indtil blokaden er ophørt.

Det er vores vurdering, at bivirkninger og risici ved deltagelse i denne undersøgelse er minimale, hvorfor det skønnes at være forbundet med meget lav risiko at deltage i forsøget.

At deltage i forsøget forventes ikke at indebære yderligere gener eller undersøgelser end ovenfor beskrevet.

Information af og samtykke fra forsøgsdeltagere

De frivillige forsøgspersoner vil blive rekrutteret ved hjælp af et opslag i det Sundhedsvidenskabelige fakultets blad for studerende, ”MOK”, i København.

De frivillige forsøgspersoner der henvender sig, vil få tilsendt et kopi af den skriftlige deltager information, og vil blive skriftlig informeret om at tage en bisidder med til den mundtlige information, hvis de ønsker dette. Forsøgspersonerne vil blive mundtligt informeret af forsøgsansvarlig læge i et aflukket lokale i en form der er forståelig for dem. Dette lokale anvendes kun til dette formål, hvorved informationen vil foregå uden unødig forstyrrelser. Der vil blive givet deltagerne den tid, de har behov for og mulighed for at få svar på spørgsmål.

Beskyttelse af data fra forsøgsdeltagere

Forsøget vil blive anmeldt til Datatilsynet og lov om behandling af personoplysninger vil blive overholdt.

Alle oplysninger vil blive behandlet fortroligt og ved indberetning af forsøgsresultater vil forsøgsdeltageren være anonyme og de ansvarlige personer for dette forsøg er underlagt tavshedspligt.

Indsamlede data i form af Case Report Form, underskrevne informerede samtykkeformularer og journal oprettet til studiet vil kun blive gjort tilgængelige for inspektion af autoriserede repræsentanter fra relevante myndigheder, inklusive Københavns Universitets Hospitals GCP enhed.

RAMMER FOR STUDIET

Tidsplan

Undersøgelsen forventes påbegyndt: 01.03.2015.
Undersøgelsen forventes afsluttet: 01.12.2015

Sted for undersøgelsens udførelse:

Anæstesiafdelingen, Køge Sygehus.

Studiedesign

**Forsøgstype:**	Kontrolleret
**Fase:**	Fase 2
**Randomisering:**	Medicin fremstillet af Skanderborg apotek i henhold til computer-genereret randomiseringsliste, ingen blok randomisering.
**Blinding:**	Blindet
**Deltagere:**	Raske forsøgspersoner
**Antal:**	21 forsøgspersoner

STUDIESELEKTION

Inklusionskriterier.

Forsøgsdeltagere skal opfylde alle følgende kriterier for at være egnede til inklusion i undersøgelsen:

- Alder $\geq$ 18 år
- Forsøgsdeltagere, som har givet deres skriftlige informerede samtykke til at deltage i undersøgelsen efter at have forstået protokollens indhold og begrænsninger fuldt ud
- ASA 1
- BMI $\geq$ 18 og $\leq$ 30
- Mænd

Eksklusionskriterier.

Forsøgsdeltagere, der opfylder et eller flere at følgende kriterier, er ikke egnede til inklusion i denne undersøgelse:

- Forsøgsdeltagere som ikke kan samarbejde til undersøgelsen.
- Forsøgsdeltagere som ikke forstår eller taler dansk.
- Allergi over for de i undersøgelsen anvendte stoffer.
- Alkoholforbrug >21 genstande per uge
- Medicinmisbrug – efter investigators skøn
- Dagligt indtag af receptpligtig smertestillende medicin indenfor de sidste 4 uger.
- Indtag af håndkøbsmedicin inden for de sidste 48 timer
- Neuromuskulære defekter, tidligere kirurgi eller andet traume på underekstremiteterne
- Diabetes Mellitus
- 2. Eller 3. Grads AV-blok
- Syg sinusknude syndrom

Økonomiske forhold

Denne undersøgelse er investigator-initieret og iværksat af Forskningsansvarlig overlæge ph.d. Ole Mathiesen, læge ph.d Pia Jæger, ledende overlæge Jørgen B. Dahl og 1. reservelæge Jakob Hessel Andersen. Udgifter i forbindelse med studiet relaterer sig til diverse anmeldelser, udstyr, forsøgsmedicinen, samt aflønning af frivillige forsøgspersoner. Forsøgspersonerne vil modtage 150,- kr per time for deltagelse i forsøget – beløbet er skattepligtigt. Alle involverede er ansat i Region Sjælland eller Region Hovedstaden. Udgifter til aflønning af forsøgspersoner og medicin dækkes af Anæstesiafdelingen, Køge Sygehus eller via en ansøgning til Regions Sjællands forskningsfond. Fonden er underlagt offentlig revision. Der ydes ingen ekstern støtte til forsøget. De forsøgsansvarlige har ingen økonomiske interesser i forsøget.

Forsøgsdeltageres gennemførelse og afbrydelse af forsøget

- En forsøgsdeltager, der har gennemført undersøgelsen, er en forsøgsdeltager, der har fulgt forsøgets behandlingsplan indtil en time efter sprit-vædet gaze igen mærkes som koldt (normal temperaturdiskrimination).
- En forsøgsdeltager, der ikke har gennemført forsøget, er en forsøgsdeltager, som inkluderes i forsøget, dvs. giver informeret samtykke og ikke gennemfører forsøget, hvad enten forsøgsdeltageren har modtaget forsøgsmedicinering eller ikke. Har en forsøgsdeltager ikke gennemført forsøget skal der redegøres for, om eller hvorledes denne følges i øvrigt i studiet, - dette gælder også dropouts – samt hvilke data der indsamles fra disse forsøgspersoner.

Årsager til forsøgsdeltagernes afbrydelse af forsøget

En forsøgsdeltager kan tages ud af forsøget under følgende omstændigheder:

- En forsøgsdeltager defineres som non-responder og tages ud af forsøget dersom der er normal temperaturdiskriminationssans for sprit-vædet gaze i et eller begge ben 2t efter blokanlæggelsen. Forsøgspersonen vil blive monitoreret for bivirkninger i 4 timer efter blokanlæggelse inden hjemsendelse.
- Hvis investigator skønner, at det vil være det bedste for forsøgsdeltageren
- Hvis forsøgsdeltageren ønsker at udgå af forsøget

Procedure for forsøgsdeltagere, der afbryder forsøget

I overensstemmelse med Helsinki Deklarationen har forsøgsdeltagerne ret til at afbryde forsøget på ethvert tidspunkt af hvilken som helst årsag. Sponser og investigator har også ret til at trække en forsøgsdeltager ud af forsøget på ethvert tidspunkt.
Årsagen til at en forsøgsdeltager tages ud af forsøget før planlagt, skal noteres i forsøgsdeltagerens Case Report Form.

METODOLOGI

Generel behandlingsplan og medicindosering

Forløb for forsøgspersonerne:

- Mundtlig information om projektet gives af forsøgsansvarlig. Der vil blive givet betænkningstid, hvis deltagerne har brug for dette
- Forsøgsansvarlig læge Jakob Hessel Andersen eller lægelig projektdeltager udfører kort journaloptagelse, hvor in- og eksklusionskriterier gennemgås, højde, vægt, BT og puls måles.
- Samtlige målemetoder afprøves, for at gøre deltagerne kendt med proceduren:
    - Temperaturdiskriminations test med spritvædet gaze
    - Pin-prick test
    - Maksimum smertescore ved tonisk varmestimulering
    - Bestemmelse af tærskelværdi for varme detektering (WDT)
    - Bestemmelse af tærskelværdi hvor varme udløser smerte (HPDT)

Derefter måles udgangsværdier for samtlige parametre.

- **Ultralydsvejledt anlæggelse af adduktor kanal blok:**
    - Blokaden anlægges ultralydsvejledt som enkelt-injektion
    - Adduktor kanal blok anlægges som bolus omkring n. saphenus i adduktorkanalen på midtlårs niveau.
    - Der anlægges først adduktor kanal blok i HØJRE ben, med indgift af behandling A eller B, i henhold til randomisering. Umiddelbart efterfølgende anlægges adduktor kanal blok i VENSTRE ben, med den modsatte behandling.
    - Alle deltagere modtager de to forskellige former for behandling, én i hvert ben.

- **Forsøgsmedicin:**

    - **Behandling A:**
      Adduktor kanal blok med 20 ml ropivacain 0,5 % + 1,0 ml clonidin 150 μg/ml

    - **Behandling B:**
      Adduktor kanal blok med 20 ml ropivacain 0,5 % + 1,0 ml isotonisk saltvand

    - **Der vil ikke planmæssigt blive givet anden medicin i forsøgsperioden**

- Tidspunktet for blokanlæggelse (fjernelse af nål) i højre ben registreres som tiden 0 (T0).
- Forsøgspersonen overvåges med blodtryk hvert 15. minut og kontinuerlig pulsoximetri og 3-punkts EKG i 4 timer efter indgift af forsøgsmedicin. Herefter hver hver time indtil sensorik er tilbage til udgangspunkt.

- Desuden vurderes patienterne på en verbal rating scale (VRS) skala 0-3 med henblik på sedation (0 ingen, 1 let, 2 moderat, 3 udtalt).
- Temperaturdiskriminationstest med spritvædet gaze, pin-prick og tonisk varmestimulering udføres til tiderne 1 og 4t postblok. Såfremt der ikke er ophævet sensorik ved t=1 udføres sensoriske tests ligeledes til t=2 og t=3, derefter hver time indtil værdierne er normaliserede/returneret til udgangsværdien. Der vil ved behov efter de første 4 timer blive mulighed for at forsøgspersonerne kan sove og sensoriske tests såvel som blodtryksmålinger (Såfremt forsøgspersonerne har stabilt blodtryk) udelades her.
- WDT og HPDT udføres efter 1t, derefter kun hvis smertescore ved tonisk varmestimulering er >0, hvorefter de udføres hver time indtil værdien er normaliseret.
- Ligeledes vil temperaturdiskriminationstest med spritvædet gaze udføres hver halve time hvis smertescore ved tonisk varmestimulering er >0.
- Den enkelte test afsluttes når værdien er returneret til udgangsværdien.
- En forsøgsdeltager defineres som non-responder og tages ud af forsøget dersom der er normal temperaturdiskriminationssans for sprit-vædet gaze og pin-prick i et eller begge ben 2t efter blokanlæggelsen.
- Et blok defineres som partielt såfremt der er nedsat sensibilitet for kulde (sprit swab) og pin-prick, men VAS>0 ved lang varmestimulering til t=2.
- Evt. bivirkninger fra tiden 0 indtil hjemsendelsen registreres i CRF
- Når alle værdier er normaliserede afsluttes forsøget og deltageren sendes hjem

Kliniske vurderinger

Generelt:

- Alle tests udføres først på højre ben og derefter på venstre ben.
- Sensoriske tests vil blive udført med deltagerne i halvt liggende stilling i et rum i afdelingen, som kun anvendes til dette.
- Testene udføres i følgende rækkefølge (dog udføres ikke alle nævnte test til alle tidspunkter): temperatur diskrimination, pin-prick og smertescore ved lang varme stimulering til sidst.
- De sensoriske tests foretages i saphenus innervations område (anteromedialt på crus). Varmestimulering udføres midt på crus, og pin-prick og temperaturdiskriminations testen udføres distalt herfor.
- Bestemmelse værdien for WDT og HPDT og måling af smerte under lang varme stimulering af huden udføres med en computerkontrolleret thermode (2,5 cm^2, Thermotest, Somedic A/B, Hörby, Sweden).
- Værdien af WDT og HPDT bestemmes ud fra fire konsekutive målinger og angives som en gennemsnitsværdi af disse. Der holdes 10-20 sekunders pause imellem hver af de konsekutive målinger.
- Smertescore ved lang varmestimulering anses som normaliseret når max VAS returnerer til udgangsværdien ± 10 mm. WDT og HPDT anses som at være normaliserede når de er returneret til udgangsværdien + 2^oC, eller derunder.

Sensorisk test:

- **Temperaturdiskriminations test:** Ved denne test ønsker vi at registrere varigheden af det sensoriske udfald, hvor stimulering af huden med sprit-vædet gaze ikke føles som kold. Varigheden defineres som tiden fra blokanlæggelsen (nålens fjernelse), indtil stimulering af huden med sprit-vædet gaze igen føles som kold.
- **Pin-prick test:** Bestemmelse af sensorisk udfald ved stimulering af huden med spids genstand (sikkerhedsnål). Ved denne test ønsker vi at registrere varigheden af det sensoriske udfald, hvor stimulering af huden med spids genstand ikke føles som spids. Denne test benyttes til at bestemme varigheden af den sensoriske blokade, defineret som tiden fra blokanlæggelsen (nålens fjernelse), indtil påvirkning af huden med spids genstand igen føles som spids.
- **Maksimum smertescore ved lang tonisk varmestimulering:** Den toniske varmestimulering består i at opvarme huden ved 45 °C i 30 sekunder. Deltageren skal vurdere smerten på en Visuel Analog Skala (VAS) hed hjælp af en VAS- lineal som i hver ende er mærket med beskrivelsen “ingen smerte” (0) og “værst mulig smerte” (100). Varigheden af det sensoriske udfald vurderet ved lang varmestimulering, defineres som tiden fra blokanlæggelsen til maksimum VAS ved lang varmestimulering er normaliseret.
- **Bestemmelse af varme-tærskelværdi (warmth detection threshold, WDT)** repræsenterer den laveste temperatur, som opfattes som varm. Starttemperatur for thermoden er 32 °C, og hastigheden for temperaturøgningen er 1 °C/sek under tærskelværdi bestemmelsen og 5 °C/sek ved retur til udgangstemperaturen efter afsluttet varmestimulering. Deltageren bedes om at trykke på en knap, for at angive tærskelværdien for varme og samtidig afslutte varme stimuleringen. Hvis grænsen for temperaturøgning på 52 °C nås før der registreres en tærskelværdi, går thermoden automatisk tilbage til sin starttemperatur og 52 °C registreres. Hvis tærskelværdien overstiger maksgrænsen på 52 °C sættes værdien til 52°C. Hver tærskelværdi beregnes som gennemsnittet af fire stimuleringer; stimuleringerne lægges med 6-10 sekunders mellemrum. Varigheden af det sensoriske udfald vurderet ved WDT, defineres som tiden fra blokanlæggelsen til WDT er normaliseret.

Bestemmelse af tærskelværdien hvor varme udløser smerte (heat pain detection threshold, HPDT) repræsenterer den laveste temperatur, som opfattes som smertefuld. Starttemperaturen for thermoden er 32 °C, og hastigheden for temperaturøgningen er 1 °C/sek og 5 °C/sek ved retur til udgangstemperaturen efter afsluttet varmestimulering. Deltageren bedes om at trykke på en knap for at angive tærskelværdien for smerte og samtidig afslutte varme stimuleringen. Hvis grænsen for temperaturøgning på 52 °C nås før der registreres en tærskelværdi, går thermoden automatisk tilbage til sin starttemperatur og 52 °C registreres. Hvis tærskelværdien overstiger maksgrænsen på 52 °C sættes værdien til 52 °C. Hver tærskelværdi beregnes som gennemsnittet af fire stimuleringer; stimuleringerne lægges med 6-10 sekunders mellemrum. Varigheden af det sensoriske udfald vurderet ved HPDT, defineres som tiden fra blokanlæggelsen til HPDT er normaliseret.
Studiet strækker sig således for hver forsøgsdeltager fra introduktionssamtalen til varigheden af blokket er ophørt, typisk 10–14 timer efter blokanlæggelsen. Forsøgsdeltageren modtager ovennævnte behandlinger i henhold til computergenereret randomiseringsliste.

MEDICIN OG MEDICINHÅNDTERING

Undersøgelsesmedicin

Aktivt præparat: **Ropivacain 5.0 mg/ml.**
Dispenseres i form af injektionsvæske i opløsning til perineural og epidural brug. Indeholder: ropivacainhydrochlorid. Endvidere natriumchlorid, saltsyre, natriumhydroxid. Vand til injektionsvæsker.

Præparatet importeret til Danmark af: Fresenius Kabi filial af Fresenius Kabi AB, Islands Brygge 57, 2300 København S

Indehaver af markedsføringstilladelsen: Fresenius Kabi AB

Markedsføringstilladelsesnummer 45008.

Aktivt præparat: **Clonidin 150 μg/ml**
Dispenseres i form af injektionsvæske i opløsning til systemisk brug. Indeholder: clonidinhydrochlorid. Endvidere saltsyre (3.6%) (Til pH neutralisering).
Vand til injektion.

Præparatet importeret til Danmark af: Specifik Pharma

Fremstiller: Boehringer Ingelheim Espana, S.A.

Markedsføringsnummer Tyskland: 6191514.00.01

Placebo: **Natriumklorid 9 mg/ml**. Infusionsvæske, opløsning. 1 l indeholder 9 g natriumchlorid i sterilt vand. Elektrolytindhold/l: 154 mmol chlorid og 154 mmol natrium. Isotonisk. Osmolaritet ca. 308 mmol/l.

Fremstiller: Skanderborg Apotek

Blindingsprocedure, pakning og etikettering

Undersøgelsen udføres som en blindet randomiseret undersøgelse. Randomiseringen vil blive foretaget ved computergenereret randomiseringsliste, af Skanderborg apotek, der i henhold til randomiseringen, fremstiller og pakker 1 kasse med forsøgsmedicin per deltager. Forsøgsmedicinen vil blive pakket og etiketteret af Skanderborg Apotek efter givne regler for dette. Den enkelte forsøgsdeltager modtager medicinen som anført under **"Generel behandlingsplan og medicindosering"**.

Alle deltager vil modtage et AKB med 20 ml ropivacain 5 mg/ml i hvert ben. Da denne behandling hverken skal blindes eller randomiseres vil medicinen blive taget fra afdelingens standardsortiment.

Clonidin 150 μg /ml og isoton natriumchlorid er begge klare farveløse væsker, identisk af udseende. Clonidin fremstilles af Boheringer Ingelheim og leveres i klare ampuller med gul

og grøn markeringsstreg. Disse ampuller fremskaffes fra producenten og der påfyldes af apoteket i disse tilsvarende identiske ampuller placebo indeholdende 1 ml isotonisk natriumchlorid. Da ampuller og indhold er identiske af udseende blindes clonidin 150 µg /ml eller placebo ved ometikkering foretaget af Skanderborg Apotek, hvorefter det ikke er muligt at se forskel på ampullerne. I hver kasse pakkes 2 stk 1,0 ml ampuller, hvoraf den ene er markeret med ”HØJRE BEN” og den anden markeret med ”VENSTRE BEN”, samt randomiseringsnummer, indeholdende enten clonidin 150 µg /ml eller placebo afhængig af randomisering. På forsøgsdagen noterer investigator batchnummer og udløb, hvorefter medicinen trækkes op i neutrale sprøjter mærket henholdsvis HØJRE BEN og VENSTRE BEN.

Der udarbejdes endvidere 2 sæt forseglede kodekuverter. Disse indeholder oplysning om, hvilken behandling den enkelte forsøgsperson er randomiseret til. Det ene sæt opbevares af sponsor, det andet opbevares af forsøgsansvarlig investigator Jakob Hessel Andersen på Anæstesiafdelingen, Køge Sygehus på et sikkert aflåst sted.

Anden behandling

Anden medicin, som anses nødvendig for forsøgspersonen, vil blive givet efter investigators skøn. Investigator er ansvarlig for alle forsøgsrelaterede medicinske beslutninger. Administration af al anden medicinsk behandling vil blive registreret på den dertil hørende Case Report Form.

Procedurer ved nødstilfælde

Investigator vil tilse, at der er procedurer og den fornødne ekspertise til at imødegå en nødsituation, som kan opstå i løbet af undersøgelsen.
Blindingen må kun brydes, hvis den fortsatte behandling af forsøgsdeltageren nødvendiggør kendskab til randomiseringskoden. Afblindingen kan foregå øjeblikkeligt såfremt det er nødvendigt og uden restriktioner, uden foregående kontakt med monitor eller sponsor.
Hvis kodekuverten åbnes, skal dato og årsag registreres, og kuverten skal signeres af investigator.

Medicinhåndtering

Alle deltager vil modtage et AKB med 20 ml ropivacain 5 mg/ml i hvert ben. Da denne behandling hverken skal blindes eller randomiseres vil medicinen blive taget fra afdelingens standardsortiment.
Al medicin vil blive håndteret af investigator. I hver kasse er der pakket to ampuller; én ampul med clonidin og én ampul med isotonisk saltvand, til indgift i henhold til markeringen i ”HØJRE BEN” eller ”VENSTRE BEN”. Investigator blander forsøgsmedicinen med 20 ml ropivacain 0,5% og indgiver medicinen i det relevante ben i henhold til markering samt randomiseringsnummer, umiddelbart efter optrækning. Begge lægemidler er klare farveløse væsker, og vil fremstå identiske af udseende og være identiske i mængde.

Medicinregnskab

Investigator vil tilse, at undersøgelsesmedicinen opbevares på et sikkert sted og kun udleveres til deltagere i denne undersøgelse. Der er kun investigator der vil håndtere forsøgsmedicinen og vil tilse at batchnummer og udløbsdato noteres (Inklusive ropivacain). Investigator gør rede for medicin, som ved et uheld eller på anden måde er bortkommet, samt enhver uoverensstemmelse mellem udleveret og returneret medicin.

BIVIRKNINGER

Bivirkninger/uønskede hændelser (AEs=adverse events)

Bivirkninger defineres som enhver uønsket hændelse, tegn eller symptomer, der optræder under deltagelse i undersøgelsen, som er tidsrelateret til administreringen af undersøgelsesmedicinen, hvad enten denne uønskede hændelse anses for at have forbindelse med undersøgelsesmedicinen eller ej. Alle uønskede hændelser skal noteres i forsøgsdeltagerens Case Report Forms. Hvis en utilsigtet hændelse opstår mere end 36 timer efter administration af undersøgelsesmedicinen, og der ikke er en tilsyneladende årsagssammenhæng eller forbindelse med undersøgelsesmedicinen, anses denne ikke for en utilsigtet hændelse.
Start og slutdato/tidspunkt, sværhedsgrad og følger efter undersøgelsesstoffet skal noteres for enhver uønsket hændelse. Sværhedsgraden af den uønskede hændelse og sammenhængen med undersøgelsesstoffet skal vurderes i overensstemmelse med nedenfor beskrevne retningslinjer.
Investigator skal vurdere sammenhæng mellem en uønsket hændelse og undersøgelsesstoffet ved hjælp af følgende retningslinjer:

Retningslinjer for uønskede hændelser eventuelle sammenhæng med behandling:

1. Ikke relateret - ingen tidsmæssig sammenhæng, andre ætiologier meget sandsynligt årsagen
2. Muligvis relateret - mindre klar sammenhæng, andre ætiologier er også mulige
3. Sandsynligvis relateret - klar tidsmæssig sammenhæng med bedring ved afbrydelse af medicinering, og ikke rimeligt forklaret ved forsøgsdeltagerens kendte kliniske tilstand.
4. Relateret - klar tidsmæssig sammenhæng med genbehandlingstest eller klinisk vurdering.

Forsøgsdeltagere, der har uønskede hændelser, vil blive monitoreret med relevante kliniske vurderinger og laboratorieundersøgelser efter behandlende læges beslutning. Alle uønskede hændelser vil blive fulgt til tilfredsstillende restituering eller stabilisering.
Ved en alvorlig uønsket hændelse (Serious Adverse Event = SAE) forstås en hændelse som medfører en betydelig risiko for død eller handikap hos forsøgsdeltageren (eller dennes afkom) inkluderende, men ikke begrænsende sig til en hændelse som resulterer i:

- død
- er livstruende – forsøgsdeltageren var efter investigators skøn i umiddelbar risiko for at dø af den uønskede hændelse, da den optrådte
- medfører hospitalsindlæggelse
- er varigt invaliderende

- er en medfødt anomali

Der vil blive anvendt produktresume for clonidin afsnit 4.8 samt produktresumeet for Ropivacain afsnit 4.8, fra Sundhedsstyrelsens hjemmeside, som referencedokument, når der skal vurderes om en alvorlig relateret bivirkning er ventet eller uventet.

Da sensoriske udfald er en velkendt følgevirkning til nerveblokader anses dette ikke som en uønsket hændelse og vil ikke indgå i rapportering til myndighederne, med mindre muskelstyrken eller følesansen mod formodning ikke skulle returnere til udgangsværdien inden 36 timer efter blok-anlæggelsen. Der er en kendt faldrisiko ved nerveblokader der involverer n.femoralis, hvorfor et fald ikke ses som en uønsket hændelse, med mindre der opstår skader i forbindelse med faldet. Risikoen for fald er formentlig lavere ved et adduktor kanal blok,[9] men der vil naturligvis blive taget alle forholdsregler for at undgå fald. Bradycardi, hypotension, sedation og svimmelhed er ligeledes kendte følgevirkninger til behandling med clonidin og vil heller ikke blive betragtet som en uønsket hændelse.

Gradering af uønskede hændelser:
Den lægelige investigator skal forsøge at finde frem til alle kliniske og objektive reaktioner fra forsøgsdeltagere i behandling og fastslå deres sammenhæng med undersøgelsesstoffet. Reaktioner, hvis der er nogen, skal graderes efter følgende skala:

1	= let
2	= moderat
3	= svær
4	= livstruende

Rapportering af AEs og SAEs bivirkninger

Investigator er ansvarlig for at alle uønskede hændelser skal registreres i forsøgsdeltagerens Case Report Form.
Sponsor er ansvarlig for den løbende overvågning af forsøgets risk/benefit forhold. Opstår eller erkendes der situationer, der kan have betydning for forsøgspersonernes sikkerhed eller forsøgets udførelse skal dette **altid** straks rapporteres til Sundhedsstyrelsen. Tilsvarende rapportering skal også ske til alle involverede investigatorer og Videnskabsetiske Komiteer.

Herudover gælder nedenstående regler for indberetning til myndigheder. Der skal indberettes hændelser og bivirkninger for al forsøgsmedicin.

Sponsor skal af investigator løbende holdes orienteret om adverse events. Ved forsøgets afslutning skal den endelige rapport indeholde en beskrivelse af alle indtrufne bivirkninger.

Serious Adverse Events – SAE øjeblikkelig indberettes af investigator til sponsor (sponsor-investigator). SAE skal af investigator indberettes én gang årligt til Videnskabsetisk komité i hele forsøgsperioden, sammen med en rapport over forsøgspersonernes sikkerhed.

Serious adverse reaction – SAR (Alvorlige, formodede bivirkninger), Skal af sponsor (sponsor-investigator) indberettes én gang årligt til Sundhedsstyrelsen. Indberetningen skal også indeholde en rapport over forsøgspersoners sikkerhed.

Suspected unexpected serious adverse reaction – SUSAR (Uventede og alvorlige formodede bivirkninger), skal af sponsor (sponsor-investigator**)** indberettes til Sundhedsstyrelsen og de videnskabsetiske komiteer. Ved rapportering til Sundhedsstyrelsen vil der blive benyttet styrelsens e-blanket til rapportering af SUSAR.

Dødelige eller livstruende SUSAR´s indberettes senest **7 dage** efter, at sponsor har fået kendskab til dem, og senest **8 dage** efter indberetningen skal sponsor meddele Sundhedsstyrelsen alle relevante oplysninger om opfølgningen.

Alle andre uventede og alvorlige formodede bivirkninger, skal indberretes til samme myndigheder senest 15 dage efter, at sponsor (sponsor-investigator) har fået kendskab til disse.

Enhver indberetning skal ledsages af kommentarer om evt. konsekvenser for forsøget.
Det anbefales endvidere at sponsor (sponsor-investigator) orienterer lægemidlets fremstiller.

STATISTISKE ANALYSER

Beregning af antal forsøgsdeltagere

Kun få tidligere studier har sammenlignet varigheden af perineural clonidin med systemisk clonidin, men disse studier har undersøgt kombination med bupivacain (4,21), mepivacain(3) og levobupivacain(22) perineuralt og ikke ropivacain. El Saied(9) undersøgte ropivacain med clonidin som adjuvans perineuralt vs. Ropivacain alene i et studie med 50 patienter. Tilsætning af clonidin som adjuvans forlængede den sensoriske blockade fra 489 til 628 minutter (95% CI 90-187). Vores gruppe har i et endnu ikke publiceret studie på raske forsøgspersoner fundet en sensorisk virkningsvarighed af AKB med 20ml ropivacain 5mg/ml på i gennemsnit 22 timer med SD 4 timer. På baggrund heraf finder vi at 240 minutters forlængelse af blokadens varighed vil være klinisk relevant. Med en type 1 fejls risiko på 5 % og en type 2 fejls risiko på 10 %, samt en SD på 240 minutter vil i alt 18 forsøgsdeltagere skulle inkluderes i dette parrede forsøg for at kunne vise en forskel på 240 minutter. For at kompensere for evt. dropouts inkluderes 21 forsøgsdeltagere.

Databearbejdning

Forsøget vil blive afsluttet når 21 forsøgspersoner er inkluderet i undersøgelsen.
Hver enkelt forsøgspersons evaluérbarhed i de statistiske analyser vil være afgjort før koden brydes. Der vil primært blive udført en intention to treat analyse. Ekskluderede forsøgspersoner samt manglende, ubenyttede eller uægte data vil blive beskrevet, og hvis relevant (For eksempel ved et partielt blok), vil der udføres en per protokol analyse.

Data vil blive opbevaret og evaluering og statistiske overvejelser analyseret i en computer, forsøgspersonens anonymitet vil blive bevaret og den lokal datalovgivning vil blive overholdt.
Fysiske data (CRF) opbevares i et aflåst skab på et aflåst kontor. Data er psudoanonymiserede da de indsamlede data registreres med løbenummer i CRF.
Omsætningsnøglen opbevares særskilt i aflåst skab på aflåst kontor. De elektroniske pseudoanonymiserede data vil blive opbevaret på en lukket mappe hvor det kun er forsøgsansvarlige der har adgang til.

Data vil blive opbevaret i 5 år efter forsøgets afslutning, hvorefter alt papirmateriale slettes ved makulering og elektroniske data anonymiseres fuldstændig. Omsætningsnøglen vil ligeledes blive slettet

Resultater vil blive beskrevet med middelværdier og standarddeviation på middelværdien, samt med medianer og interkvartiler. Der anvendes 5 % signifikansniveau. Kontinuerlige data der er normalfordelte vil blive sammenlignet med en parret t-test, medens Wilcoxon signed-rank test vil blive brugt til non-parametriske data. Ved ændring af den statistiske plan vil der blive redegjort herfor ved publicering.

DATAREGISTRERING, SAMT REGLER FOR KONTROL AF UNDERSØGELSESPROCEDURER

Undersøgelsen skal gennemføres i overensstemmelse med de gældende regler for kliniske forsøg der omfatter mennesker vedrørende kvalitetskontrol og kvalitetsstyring, og vil følge Good Clinical Practice - guidelines.
Investigator og medinvestigatorer på Køge Sygehus er ansvarlige for håndtering og arkivering af data efter gældende regler. Data tilhører investigator Jakob Hessel Andersen, Anæstesi afdelingen, Køge Sygehus, og sponsor Ole Mathiesen, Anæstesi afdelingen Køge Sygehus.

Case Report Forms

For hver forsøgsdeltager inkluderet i undersøgelsen vil en Case Report Form (CRF) blive udfyldt. Denne vil blive signeret af investigator for at bekræfte rigtigheden af data. Rettelser af data vil kun blive foretaget ved at strege de forkerte data ud (den ukorrekte information vil forblive synlig og læselig) og de korrekte data vil blive skrevet ved siden af de udstregede. Korrektionslak vil ikke blive anvendt. Rettelser vil blive dateret og signeret af investigator eller dennes stedfortræder.
Sourcedata er defineret som værende: CRF.

Uddannelse

Investigator vil sikre, at det involverede personale er passende uddannet og instrueret og har de fornødne oplysninger til udførelse af undersøgelsen.

YDERLIGERE KRAV OG GENEREL INFORMATION

Forsikring

For frivillige forsøgspersoner er gældende at med hensyn til enhver skade, forårsaget direkte eller indirekte af undersøgelsesmedicinen i denne kliniske undersøgelse, påtager anæstesiologisk afdeling Køge Sygehus sig det lovmæssige ansvar på investigator og dennes medarbejderes vegne, forudsat at investigator og dennes medarbejdere har fulgt de instruktioner, som er givet i denne protokol samt eventuelle tillæg dertil, samt at investigator og dennes medarbejdere har udført undersøgelsen videnskabeligt og i overensstemmelse med gældende regler og accepterede teknikker. Forsøgsdeltagerne er i tilfælde af skade eller død uden sammenhæng med undersøgelsens gennemførelse forsikret af hospitalets forsikring.

Offentliggørelse af resultater
På basis af data vil investigator skrive en rapport over undersøgelsen. Denne rapport vil blive fremsendt til relevante myndigheder. Rapporten vil også danne basis for et manuskript til publikation med følgende forfatter rækkefølge:

1. **Jakob Hessel Andersen**
2. **Pia Jæger**
3. **Tobias Laier Sonne**
4. **Ole Mathiesen**
5. **Jørgen B. Dahl**
6. **Ulrik Grevstad**

Forsøgets resultater, såvel negative som positive, og inkonklusive forsøgsresultater vil under alle omstændigheder blive offentliggjort.
