# Supplementary material for: Clonidine used as a perineural adjuvant to ropivacaine, does not prolong the duration of sensory block when controlling for systemic effects: A paired, blinded, randomized trial in healthy volunteers
Source: PLoS One. 2017 Sep 7;12(9):e0181351. doi: 10.1371/journal.pone.0181351 (PMC5589088; doi:10.1371/journal.pone.0181351)
Supplement: S2 Text — (PDF) [file pone.0181351.s002.pdf]

Does Perineural Clonidine Prolong the Duration of an Adductor Canal Block When Controlling for Possible Systemic Effects?
- A Randomized Paired Trial in Healthy Volunteers
SM1-JH-14, EudraCT 2014-005640-18, VEK nr SJ-437

PROTOCOL

" Does Perineural Clonidine Prolong the Duration of an Adductor Canal Block When Controlling for Possible Systemic effects? - A Randomized Paired Trial in Healthy Volunteers"

Primary investigator:
Name: **Jakob Hessel Andersen**
Title: **MD**

Sub investigators:
Name: **Pia Jæger**
Title: **MD**

Name: **Tobias Laier Sonne**
Title: **MD**

Name: **Jørgen B. Dahl**
Title: **MD**

Name **Ulrik Grevstad**
Title **MD**

Sponsor:
Name: **Ole Mathiesen**
Title: **MD**

- **Sponsor**:
  Ole Mathiesen
  Senior consultant, PhD, Clinical research associate professor
  Department of Anesthesiology
  University hospital Zealand, Køge
  Lykkebækvej 1
  4600 Køge
  Phone: +45 42376321/+45 23462485
  Email: Omat@Regionsjaelland.dk

- **Primary investigator:**
  Jakob Hessel Andersen
  Staff specialist
  Department of Anesthesiology
  University hospital Zealand, Køge
  Lykkebækvej 1
  4600 Køge
  Denmark
  Phone: +45 47326321/+45 60610666
  Email: Jahea@regionsjaelland.dk.dk

- **Sub investigator:**
  Tobia Laier Sonne
  MD
  Department of Anesthesiology
  University hospital Zealand, Køge
  Lykkebækvej 1
  4600 Køge
  Denmark
  Phone: +45 24879182
  Email: TOBS@regionsjaelland.dk

- **Sub investigator:**
  Pia Jæger
  MD, PhD
  Department of Anesthesiology
  Section 4231
  Rigshospitalet, Blegdamsvej 9
  2100 Copenhagen Ø
  Denmark
  Phone: +45 3024 3924
  Email: pia.therese.jaeger@regionh.dk

- **Sub investigator**
  Jørgen B. Dahl
  Head of Department, DMSc, MBAex
  Department of Anesthesiology, Bispebjerg Hospital
  Bispebjerg Bakke 23
  2400 København NV
  Phone: +45 21379772
  Email: jbdahl@dadlnet.dk

- **Sub investigator:**
  Ulrik Grevstad
  Senior Consultant, PhD
  Department of Anesthesiology
  Gentofte Hospital
  Kildegårdsvej 28
  2900 Hellerup
  Denmark
  Phone.: +45 38673892
  Email: ulrik.grevstad@hotmail.com

- **Monitor:**
  GCP unit at Copenhagen University Hospital
  Bispebjerg Hospital, building 51, 3rd floor
  Bispebjerg Bakke 23
  2400 Copenhagen NV
  Phone: 3531 3887

- **Pharmacy collaborator:**
  Pharmacy of Skanderborg
  Adelgade 27
  8660 Skanderborg

" Does Perineural Clonidine Prolong the Duration of an Adductor Canal Block When Controlling for Possible Systemic Effects? - A Randomized Paired Trial in Healthy Volunteers "

Date

The sponsor confirms to adhere to the contents of the protocol and conduct this trial according to the rules and regulations of Good Clinical Practice.

- **<u>Sponsor</u>**:
  Ole Mathiesen
  Senior consultant, PhD
  Department of Anesthesiology
  University hospital Zealand, Køge
  Lykkebækvej 1
  4600 Køge, Denmark

Signature and date: ___________________________

The primary investigator hereby commits to oblige to this protocol at any given time and adhere to the rules of Good Clinical practice.

- **<u>Primary investigator:</u>**
  Jakob Hessel Andersen
  Staff specialist
  Department of Anesthesiology
  University hospital Zealand, Køge
  Lykkebækvej 1
  4600 Køge, Denmark

Date: ___________________________

PROTOCOLTITLE 1

CONTACT INFORMATION OF INVESTIGATORS 2

INTRODUCTION AND RATIONALE 7
BACKGROUND 7
LITTERATURE 8
AIM OF TRIAL 11
END POINTS 11
PRIMARY END POINT 11
SECONDARY END POINTS 11

ETHICAL CONSIDERATIONS 11
INFORMATION AND INFORMED CONSENT OF THE PARTICIPANTS 12
PROTECTION OF DATA OF THE PARTICIPANTS 14

TRIAL SETTINGS 14
TIMEFRAME 14
PLACE OF CONDUCT OF THE TRIAL 15
TRIAL DESIGN 15

TRIAL SELECTION 15
INCLUSION CRITERIA 15
EXCLUSION CRITERIA 15
ECONOMICS 16
PARTICIPANTS COMPLETION AND INTERUPTION OF TRIAL 16
REASONS FOR INTERUPTION OF TRIAL 16
PROCEDURE FOR INTERUPTION OF TRIAL 16

METHODOLOGY 17
TREATMENT PLAN AND MEDICAL DOSAGE 17
CLINICAL ASSESSMENTS 18

MEDICINE AND HANDLING 19
TRIAL MEDICATION 19
BLINDINGPROCEDURE, PACKAGING AND LABELING 20
ADDITIONAL TREATMENT 21
EMERGENCY PROCEDURE 21
MEDICINE RECORDS 21

SIDE EFFECTS 21
AES=ADVERSE EVENTS 22

REPPORTING AES AND SERIOUS AES 23

STATISTICAL ANALYSIS 24
SAMPLE SIZE CALCULATION 24
DATA HANDLING 24

DATAREGISTRATION AND RULES OF CONTROL OF PROCEDURES 24

CASE REPORT FORMS 25
EDUCATION 25

FORMAL DEMANDS AND GENERAL INFORMATION 25
INSURRANCE 25

INTRODUCTION

Background

Peripheral nerve blocks are frequently used as part of a multimodal pain treatment. The advantages of nerve blocks are, in addition to pain relief, reduced morphine consumption and consequently less morphine-related side effects such as nausea, vomiting and sedation. Nerve blocks can be performed either as a single (bolus) or as a continuous infusion using perineural catheters. The limitation of single-shot blocks is the relatively short duration. Compared to the catheter-based solutions single-shot blocks may be associated with a lower risk of infection, are considered to be less complicated to perform and can easily be used for outpatients (1, 2).

In order to prolong the effect of single-shot blockades, attempts have been made to add various adjuvants for local anesthetics with varying success. Several studies have shown that clonidine prolongs the duration of a single-shot nerve block compared to placebo (3-20), but it is unclear whether this effect is exerted via a systemic or local mechanism (3-5, 21-23). We wish to investigate whether perineural administration of clonidine prolongs the duration of a ropivacaine nerve block, when controlling for systemic effects.

Our group has validated different methods that can be used to assess the duration of sensory block, and found that the following methods can estimate the duration with high precision: temperature discrimination test with an alcohol swab, pin-prick, warmth detection threshold, WDT, and heat pain detection threshold, HPDT, as well as pain during tonic heat stimulation of the skin (24).

Clonidines mechanism of action will be investigated using an adductor canal block (ACB). This is a peripheral nerve block which has been found to be effective in treating postoperative pain after knee alloplastic surgery, with the advantage of being an almost purely sensory block (25-27). The trial will be conducted as a randomized, blinded, paired trial in healthy volunteers. All participants will receive bilateral ACB with 20 mL 0.5% ropivacaine in each thigh. According to the randomization 1.0 ml of clonidine 150 ug/ml will be added to the local anesthetic on one side and 1.0 ml of placebo (normal saline) on the opposite side.
There is a dose response relationship between clonidine and duration of the nerve block. Most studies with positive results have used a clonidine dose of 150 µg.

Nerve Blocks are a well-established treatment for surgery and pain relief, and a follow-up study of patients after receiving adductor channel blocks showed no signs of nerve damage 3-6 months after surgery (28). The models used in the study is validated in other medical specialties, and the discomfort experienced is normally only perceived as mild.
A study on isolated sensory neurons from rats showed that clonidine was not associated with increased cell death when used as an adjuvant for ropivacaine (29).

The aim of the trial is to investigate if clonidine used as an adjuvant to ropivacaine prolongs the duration of a single-shot nerve block by a peripheral mechanism when controlling for systemic effects. Our hypothesis is, that the perineural injection of clonidine as an adjuvant to ropivacaine prolongs the duration of an ACB compared to ropivacaine + placebo.

References

1. Cuvillon P, Ripart J, Lalourcey L, Veyrat E, L'Hermite J, Boisson C, et al. The continuous femoral nerve block catheter for postoperative analgesia: bacterial colonization, infectious rate and adverse effects. Anesth Analg [Internet]. 2001 Oct [cited 2014 Nov 6];93(4):1045–9. Available from: http://www.ncbi.nlm.nih.gov/pubmed/11574381

2. Liu SS, Salinas F V. Continuous plexus and peripheral nerve blocks for postoperative analgesia. Anesth Analg [Internet]. 2003 Jan [cited 2014 Nov 6];96(1):263–72. Available from: http://www.ncbi.nlm.nih.gov/pubmed/12505964

3. Singelyn FJ, Dangoisse M, Bartholomée S, Gouverneur JM. Adding clonidine to mepivacaine prolongs the duration of anesthesia and analgesia after axillary brachial plexus block. Reg Anesth [Internet]. [cited 2014 Nov 6];17(3):148–50. Available from: http://www.ncbi.nlm.nih.gov/pubmed/1606097

4. Barioni MFG, Lauretti GR, Lauretti-Fo A, Pereira NL. Clonidine as coadjuvant in eye surgery: comparison of peribulbar versus oral administration. J Clin Anesth [Internet]. 2002 Mar [cited 2014 Nov 6];14(2):140–5. Available from: http://www.ncbi.nlm.nih.gov/pubmed/11943529

5. Iskandar H, Benard A, Ruel-Raymond J, Cochard G, Manaud B. The analgesic effect of interscalene block using clonidine as an analgesic for shoulder arthroscopy. Anesth Analg [Internet]. 2003 Jan [cited 2014 Nov 6];96(1):260–2, table of contents. Available from: http://www.ncbi.nlm.nih.gov/pubmed/12505963

6. Eledjam JJ, Deschodt J, Viel EJ, Lubrano JF, Charavel P, d'Athis F, et al. Brachial plexus block with bupivacaine: effects of added alpha-adrenergic agonists: comparison between clonidine and epinephrine. Can J Anaesth [Internet]. 1991 Oct [cited 2014 Nov 6];38(7):870–5. Available from: http://www.ncbi.nlm.nih.gov/pubmed/1742820

7. Singelyn FJ, Gouverneur JM, Robert A. A minimum dose of clonidine added to mepivacaine prolongs the duration of anesthesia and analgesia after axillary brachial plexus block. Anesth Analg [Internet]. 1996 Nov [cited 2014 Nov 6];83(5):1046–50. Available from: http://www.ncbi.nlm.nih.gov/pubmed/8895283

8. Bernard JM, Macaire P. Dose-range effects of clonidine added to lidocaine for brachial plexus block. Anesthesiology [Internet]. 1997 Aug [cited 2014 Nov 6];87(2):277–84. Available from: http://www.ncbi.nlm.nih.gov/pubmed/9286891

9. El Saied AH, Steyn MP, Ansermino JM. Clonidine prolongs the effect of ropivacaine for axillary brachial plexus blockade. Can J Anaesth [Internet]. 2000 Oct [cited 2014 Nov 6];47(10):962–7. Available from: http://www.ncbi.nlm.nih.gov/pubmed/11032270

10. Erlacher W, Schuschnig C, Koinig H, Marhofer P, Melischek M, Mayer N, et al. Clonidine as adjuvant for mepivacaine, ropivacaine and bupivacaine in axillary, perivascular brachial plexus block. Can J Anaesth [Internet]. 2001 Jun [cited 2014 Nov 6];48(6):522–5. Available from: http://www.ncbi.nlm.nih.gov/pubmed/11444444

11. Iskandar H, Guillaume E, Dixmérias F, Binje B, Rakotondriamihary S, Thiebaut R, et al. The enhancement of sensory blockade by clonidine selectively added to mepivacaine after midhumeral block. Anesth Analg [Internet]. 2001 Sep [cited 2014 Nov 6];93(3):771–5. Available from: http://www.ncbi.nlm.nih.gov/pubmed/11524354

12. Adnan T, Elif AA, Ayşe K, Gülnaz A. Clonidine as an adjuvant for lidocaine in axillary brachial plexus block in patients with chronic renal failure. Acta Anaesthesiol Scand [Internet]. 2005 Apr [cited 2014 Nov 6];49(4):563–8. Available from: http://www.ncbi.nlm.nih.gov/pubmed/15777307

13. Iohom G, Machmachi A, Diarra D-P, Khatouf M, Boileau S, Dap F, et al. The effects of clonidine added to mepivacaine for paronychia surgery under axillary brachial plexus block. Anesth Analg [Internet]. 2005 Apr [cited 2014 Nov 6];100(4):1179–83. Available from: http://www.ncbi.nlm.nih.gov/pubmed/15781541

14. Reinhart DJ, Wang W, Stagg KS, Walker KG, Bailey PL, Walker EB, et al. Postoperative analgesia after peripheral nerve block for podiatric surgery: clinical efficacy and chemical stability of lidocaine alone versus lidocaine plus clonidine. Anesth Analg [Internet]. 1996 Oct [cited 2014 Nov 6];83(4):760–5. Available from: http://www.ncbi.nlm.nih.gov/pubmed/8831317

15. Casati A, Magistris L, Fanelli G, Beccaria P, Cappelleri G, Aldegheri G, et al. Small-dose clonidine prolongs postoperative analgesia after sciatic-femoral nerve block with 0.75% ropivacaine for foot surgery. Anesth Analg [Internet]. 2000 Aug [cited 2014 Nov 6];91(2):388–92. Available from: http://www.ncbi.nlm.nih.gov/pubmed/10910854

16. Madan R, Bharti N, Shende D, Khokhar SK, Kaul HL. A dose response study of clonidine with local anesthetic mixture for peribulbar block: a comparison of three doses. Anesth Analg [Internet]. 2001 Dec [cited 2014 Nov 6];93(6):1593–7, table of contents. Available from: http://www.ncbi.nlm.nih.gov/pubmed/11726451

17. Bharti N, Madan R, Kaul HL, Khokhar SK, Mishra S. Effect of addition of clonidine to local anaesthetic mixture for peribulbar block. Anaesth Intensive Care [Internet].

2002 Aug [cited 2014 Nov 6];30(4):438–41. Available from: http://www.ncbi.nlm.nih.gov/pubmed/12180581

18. Naja ZM, Ziade FM, El-Rajab MA, Naccash N, Ayoubi J-M. Guided paravertebral blocks with versus without clonidine for women undergoing breast surgery: a prospective double-blinded randomized study. Anesth Analg [Internet]. 2013 Jul [cited 2014 Nov 6];117(1):252–8. Available from: http://www.ncbi.nlm.nih.gov/pubmed/23632052

19. Yazbeck-Karam VG, Siddik-Sayyid SM, Abi Nader EL, Barakat DE, Karam HS, Cherfane GM, et al. Supplementation of retrobulbar block with clonidine in vitreoretinal surgery: effect on postoperative pain. J Clin Anesth [Internet]. 2011 Aug [cited 2014 Nov 6];23(5):393–7. Available from: http://www.ncbi.nlm.nih.gov/pubmed/21802630

20. YaDeau JT, LaSala VR, Paroli L, Kahn RL, Jules-Elysée KM, Levine DS, et al. Clonidine and analgesic duration after popliteal fossa nerve blockade: randomized, double-blind, placebo-controlled study. Anesth Analg [Internet]. 2008 Jun [cited 2014 Nov 6];106(6):1916–20. Available from: http://www.ncbi.nlm.nih.gov/pubmed/18499632

21. Culebras X, Van Gessel E, Hoffmeyer P, Gamulin Z. Clonidine combined with a long acting local anesthetic does not prolong postoperative analgesia after brachial plexus block but does induce hemodynamic changes. Anesth Analg [Internet]. 2001 Jan [cited 2014 Nov 6];92(1):199–204. Available from: http://www.ncbi.nlm.nih.gov/pubmed/11133627

22. Mannion S, Hayes I, Loughnane F, Murphy DB, Shorten GD. Intravenous but not perineural clonidine prolongs postoperative analgesia after psoas compartment block with 0.5% levobupivacaine for hip fracture surgery. Anesth Analg [Internet]. 2005 Mar [cited 2014 Nov 6];100(3):873–8, table of contents. Available from: http://www.ncbi.nlm.nih.gov/pubmed/15728081

23. Pratap JN, Shankar RK, Goroszeniuk T. Co-injection of clonidine prolongs the anesthetic effect of lidocaine skin infiltration by a peripheral action. Anesth Analg [Internet]. 2007 Apr [cited 2014 Nov 6];104(4):982–3. Available from: http://www.ncbi.nlm.nih.gov/pubmed/17377118

24. Jæger P, Grevstad U, Henningsen MH, Gottschau B, Mathiesen O, Dahl JB. Effect of adductor-canal-blockade on established, severe post-operative pain after total knee arthroplasty: A randomised study. Acta Anaesthesiol Scand. 2012;56:1013–9.

25. Jenstrup MT, Jæger P, Lund J, Fomsgaard JS, Bache S, Mathiesen O, et al. Effects of Adductor-Canal-Blockade on pain and ambulation after total knee arthroplasty: A randomized study. Acta Anaesthesiol Scand. 2012;56:357–64.

26. Jaeger P, Nielsen ZJK, Henningsen MH, Hilsted KL, Mathiesen O, Dahl JB. Adductor canal block versus femoral nerve block and quadriceps strength: a randomized, double-blind, placebo-controlled, crossover study in healthy volunteers. Anesthesiology. 2013;118:409–15.

27. Henningsen MH, Jaeger P, Hilsted KL, Dahl JB. Prevalence of saphenous nerve injury after adductor-canal-blockade in patients receiving total knee arthroplasty. Acta Anaesthesiol Scand [Internet]. 2013;57:112–7. Available from: http://www.ncbi.nlm.nih.gov/pubmed/23074997

28. Williams BA, Hough KA, Tsui BYK, Ibinson JW, Gold MS, Gebhart GF. Neurotoxicity of adjuvants used in perineural anesthesia and analgesia in comparison with ropivacaine. Reg Anesth Pain Med [Internet]. [cited 2014 Oct 27];36(3):225–30. Available from: http://www.pubmedcentral.nih.gov/articlerender.fcgi?artid=3085859&tool=pmcentrez&rendertype=abstract

29. McCartney CJL, Duggan E, Apatu E. Should we add clonidine to local anesthetic for peripheral nerve blockade? A qualitative systematic review of the literature. Reg Anesth Pain Med [Internet]. [cited 2014 Nov 6];32(4):330–8. Available from: http://www.ncbi.nlm.nih.gov/pubmed/17720118

Purpose

The purpose of the trial is to investigate whether clonidine used as an adjuvant to ropivacaine prolongs block duration by a peripheral mechanism when controlling for systemic effects. Our hypothesis is that perineural coadministration of clonidine and ropivacaine prolongs the duration of an ACB compared with ropivacaine and placebo.

Outcomes

Primary outcome measure

- The difference in duration of sensory block between ACB with ropivacaine + clonidine versus ACB with ropivacaine + placebo, measured as time from block performance (removal of the needle) until an alcohol swab feels cold again (temperature discrimination).

Secondary outcome measures

- The difference in duration of sensory block between ACB with ropivacaine + clonidine versus ACB with ropivacaine + placebo, measured as time from block performance (removal of the needle) until the stimulation with a needle again elicits a painful response (feels sharp).

- The difference in duration of sensory block between ACB with ropivacaine + clonidine versus ACB with ropivacaine + placebo, measured as time from block performance (removal of the needle) until pain during tonic heat stimulation returns to baseline values (± 10 mm on a Visual Analogue Scale (VAS)).
- The difference in duration of sensory block between ACB with ropivacaine + clonidine versus ACB with ropivacaine + placebo, measured as time from block performance (removal of the needle) until warmth detection threshold (WDT) returns to baseline values (+2° C or below).
- The difference in duration of sensory block between ACB with ropivacaine + clonidine versus ACB with ropivacaine + placebo, measured as time from block performance (removal of the needle) until pain heat pain detection threshold (HPDT) returns to baseline values (+2° C or below).
- The difference in maximum pain score (VAS) during tonic heat stimulation of the skin (30 seconds at 45 ° C) between ACB with ropivacaine + clonidine versus ACB with ropivacaine + placebo, at 4 hours post block.
- The difference in maximum pain score (VAS) during tonic heat stimulation of the skin (30 seconds at 45 ° C) between ACB with ropivacaine + clonidine versus ACB with ropivacaine + placebo, at 1 hour after resolution of the block (rebound pain).

Ethical considerations

General considerations

Ropivacaine is a well-established local anesthetic registered for perineural use, and is administered within recommended doses.

Clonidine is approved for systemic use. Clonidine is not approved for perineural administration, but is well–established in the literature as an adjuvant for peripheral nerve blocks. There are no existing reports of permanent nerve damage in studies employing perineural clonidine, and an in vitro study involving rats did not find increased incidence of nerve inflammation/damage when clonidine was coadministered with ropivacaine compared to ropivacaine alone. It is therefore our opinion that side effects and risks associated with participation in this trial are minimal.
The nerve blocks are performed using ultrasound guidance minimizing the risk of complications. The nerve blocks are performed by one anesthesiologists trained in ultrasound-guided nerve blocks.

The pain-induction model applied in this trial is simple, safe and reproducible.

This trial is conducted in healthy volunteers, since a paired bilateral design is necessary to control for systemic effects.

In this trial, we wish to test the hypothesis that clonidine used as an adjuvant to the ropivacaine in a peripheral nerve block extends the duration of the block by a local mechanism, compared to ropivacaine + placebo. The participants will not benefit directly from the trial, but the results of the trial may become a benefit for patients in the future. We are investigating if clonidine prolongs the duration of a nerve block by a peripheral mechanism. If no

effect is seen, then ineffective treatment can be avoided in the future. A demonstrated peripheral effect can lead to a more efficient postoperative pain treatment.

The trial will be conducted according to the Declaration of Helsinki principles. The protocol will be sent to the Regional Ethics Committee, The National Department of Health and the Danish Data Protection Agency. The principal investigator will inform the Regional Ethics Committee, The National Department of Health and the Danish Data Protection Agency in case of significant changes to the protocol. The trial will be registered at https://eudract.ema.europa.eu and https://www.clinicaltrials.gov.

Risks, side effects and disadvantages

All procedures, including peripheral nerve blocks, are associated with possible complications, albeit very rarely (0.02-0.4%).

Known complications are:

- Nerve injury (incidence 0.04%). Approximately 99 % of all nerve injuries resolves without treatment within one year.
- Used correctly, no side effects are seen associated with ropivacaine, but upon accidental intravascular injection, serious toxic side effects such as paresthesia, convulsions and circulatory collapse may occur. The risk of intravasal injection of the local anesthetic is minimal, and is reduced by the use of ultrasound and thorough aspiration prior to and after every 5 ml of local anesthetic.
- Systemic side effects of clonidine are transient and often mild. Sedation is normally not perceived as unpleasant. Hypotension (MAP <55) can be treated by administering isotonic saline or ephedrine 10 mg. Bradycardia can occur, but rarely requires treatment. Significant bradycardia of HR<40 can be treated with atropine 0.5 mg iv.
- A small hematoma can occur at the needle puncture site. This may cause tenderness but resolves without treatment.

The nerve blocks will be performed using ultrasound guidance, which probably reduces the risk of complications.

There is a dose response relationship between clonidine and the prolongation of the nerve block. Most studies with positive results used a dose of 150 µg, and at the same time reports a minimum of side effects (30). Clonidine is used frequently daily at the hospital for the treatment of shivering on recovery wards after surgery and opioid abstinence in the ICU. At doses of 300 micrograms, more significant sedation and hemodynamic instability is observed (30).

A peripheral venous catheter is inserted, which rarely leads to a stinging sensation and a small hematoma after removal.

A previous trial has shown, that after the performance of an ACB, a minor motor block that will not bother the subject's mobility may be observed(27). The participants will be observed until the block resolves.

It is our opinion, that risks and side effects in relation to this study is minimal, and that participation is associated with very low risk. Participation in this trial is not expected to be associated with any other nuisance, besides the already mentioned above.

Informed consent from participants

The participants will be recruited from the news bulletin for medical students ”MOK” in Copenhagen.

The volunteers will be screened in a telephone interview. A copy of the participant trial information will be send prior to the trial date. They will be informed about the possibility of bringing an external assessor. On the trial date, participants will receive oral information by the principal investigator in a closed room without interruptions, in a language understandable to them. Sufficient time for questions will be provided.

Data protection

The trial will be registered at the Danish Data Protection Agency and be conducted in accordance with the data protection policies.

All data will be handled confidentially and all reported data is anonymized. The trial investigators are subject to patient/doctor confidentiality.

The collected data in the form of a Case Report Form and the signed informed consent will only be made accessible for inspection to authorized representatives from the competent authority including members of Good Clinical Practice Unit at the University Hospital Copenhagen.

Trial setup:

Timeframe

The trial is expected to start 01.03.2015.
The trial is expected to end 01.12.2015

Trial location:

Department of Anesthesiology, Køge Hospital

Study design

**Trial type:**	Controlled
**Phase:**	Phase 2
**Randomization:**	Medicine is prepared by the pharmacy in accordance with a computer-generated randomization list (no block randomization).
**Blinding:**	Blinded
**Participants:**	Healthy volunteers
**Number:**	21 participants

Trial Selection

Inclusion criteria

Participants must meet all of the following criteria in order to be included in the trial:

- Age ≥ 18 years
- Participants must understand the protocol fully and sign the written informed consent.
- ASA 1
- BMI ≥ 18 to ≤ 30
- Male

Exclusion criteria

Participants meeting one or more of the following criteria cannot be included in the trial:

- Participants unable to cooperate in the trial.
- Participants unable to speak or read Danish
- Allergy to study medication.
- Alcohol consumption >21 units per week
- Daily intake of prescription painkillers within the last 4 weeks.
- Over the counter painkillers during the last 48 hours.
- Neuromuscular defects, former surgery or trauma to the lower extremities.
- Diabetes Mellitus
- 2. or 3.rd degree heart block
- Sick sinus node.

Economy

This trial is investigator initiated by clinical research associate professor, MD, Ph.D. Ole Mathiesen, MD, Ph.D. Pia Jæger, Head of department, MD, DMSc, Jørgen B. Dahl and staff specialist MD Jakob Hessel Andersen. The expenditure related to the trial relates to registration fees, equipment, trial medication and salary for the healthy volunteers. The participants will receive 150 DKK per hour for participating – The amount is taxable. Region Zealand or Region Hovedstaden employs all the involved personnel. The expenditure related to the trial (salary for the participants and trial medication) is covered by a grant from Region Zealand Research Foundation and the department of Anesthesiology University Hospital Zealand, Køge. The investigators have no economic interests regarding the trial.

Participants completion and interruption of trial

- A participant has completed the trial, when the treatment plan has been followed until one hour after the alcohol-swab again is perceived as cold (normal temperature discrimination).
- A participant who interrupts the trial, is a participant who is included in the trial and has given written informed consent, but has not completed the trial, whether trial medication was given or not. If a participant does not complete the trial, or drops out, a follow-up plan must be stated on if and how data is collected.

Reasons for trial interruption

A participant can be withdrawn from the trial under the following circumstances:

- A participant is defined as non-responder and withdrawn from the trial if temperature discrimination is present, when stimulated with an alcohol swab in one or both legs 2 hours post block. The participant will be monitored for adverse events for 4 hours after block performance before discharge.
- If the investigator finds it best for the participant.
- If the participant wishes to interrupt the trial.

Procedure for participants who drops out or in other way interrupts the trial

Participants have the right to withdraw from the trial at any given time and for whatever reason in accordance with the Declaration of Helsinki. Sponsor and investigator also have the right to withdraw a participant at any given time.
The reason for withdrawal will be stated in the participants Case Report Form.

METHODOLOGY

General treatment plan and medical dosing

Trial schedule for the participants:

- Information about the project is given by the principal investigator. Time for questions and consideration will be provided.
- The principal investigator Jakob Hessel Andersen (MD) or sub investigator (MD) obtains a short medical history according to the Case Report Form going through in- and exclusion criteria. Height, weight Blood pressure and Pulse are measured.
- All sensory and motor modalities are tested on the participants to familiarize them with the procedures.
    - Temperature-discrimination test with an alcohol swab
    - Pin-prick test
    - Pain during tonic heat stimulation (baseline value is measured)
    - Warmth detection threshold (WDT)
    - Heat pain detection threshold

Baseline measurements are done for all modalities.

- **Ultrasound guided ACB:**
    - The ACB is performed using ultrasound guidance as a single injection of local anesthetics next to the saphenous nerve in the adductor canal at a mid-thigh level.
    - The ACB´s are performed on the RIGHT side first followed immediately by the opposite treatment on the LEFT side according to randomization.
    - All participants receive both treatments, one in each leg.

- **Trial medication:**

    - **Treatment A:** ACB with 20 ml ropivacaine 0,5 % + 1,0 ml clonidine 150 µg/ml
    -
    - **Treatment B:** ACB with 20 ml ropivacaine 0,5 % + 1,0 ml isotonic saline

    - **No other medication is planned during the trial period.**

    - Time of block performance (removal of needle) in the right arm is registered as T=0.

- The participants are monitored with continuous ECG, pulse oximetry and blood pressure every 15 minutes for 4 hours after administration of the trial medication.
- Sedation is measured every hour post block using a verbal rating scale (VRS) from 0-3 (0 no sedation, 1 light sedation, 2 moderate sedation, 3 pronounced sedation).
- Temperature discrimination test with an alcohol swab, pin-prick and tonic heatstiemulation is performed at T=1h and 4h post block. If temperature discrimination is retained at t = 1h the sensory tests are also done at t = 2h and t = 3h. From 4 h

and forth the tests are done every hour until values are normalized/returned to baseline. If necessary after the first 4 hours there will be given opportunity to sleep and sensory tests as well as blood pressure measurements (if subjects have stabile blood pressure) is omitted here.

- WDT and HPDT are performed at T=1h. If pain score during tonic heat stimulation equals 0 WDT and HPDT are omitted. When pain score during tonic heat stimulation again is > 0, WDT and HPDT are performed every hour until the values have normalized.
- Similarly, temperature discrimination testing with an alcohol swab is performed every half hour if the pain score during tonic heat stimulation is > 0.
- The individual test finishes when the value has returned to baseline.
- A participant is defined as a non-responder and withdrawn from the trial if normal temperature discrimination and pin-prick persists in one or both legs 2h after block performance.
- A block is defined as partial if there is a reduced sensitivity to cold (alcohol swab) and pin-prick, but VAS > 0 tonic heat stimulation at t = 2h.
- Side effects from t=0 until discharge is recorded in the CRF.
- When all values are normalized the trial is terminated and the participant sent home.

Clinical assessments

Generally:

- All tests are done on the right leg first, followed by the left leg.
- Sensory tests will be performed with the participants in a semi-recumbent position in a room solely used for the trial.
- The tests are done in the following order (all tests are not necessarily done at all times): Temperature discrimination test, Pinprick, WDT, HPDT and Pain during tonic heat stimulation.
- The sensory tests are done on the anteromedial aspect of the lower leg (crus) in the area supplied by the saphenous nerve.
- WDT, HPDT and Pain during tonic heat stimulation is measured using a computer controlled thermode (2,5 cm², Thermotest, Somedic A/B, Hörby, Sweden).
- WDT and HPDT are determined from the average of four consecutive measurements. A 10-20 second pause will be held between each of the consecutive measurements.
- Pain during tonic heat stimulation is considered normalized when max VAS returns to baseline ± 10 mm. WDT and HPDT are considered normalized when returned to baseline + 2 ° C or less.

Sensory tests:

- **Temperature discrimination test:** With this test, we wish to register the duration of sensory nerve block where the stimulation of the skin with an alcohol swab does not feel cold. The duration of the nerve block is defined as time from block performance (removal of the needle) until stimulation of the skin with an alcohol swab feels cold again.

- **Pinprick test:** Determination of duration of sensory nerve block defined as the inability to distinguish blunt from sharp when stimulated with a needle. This test determines the duration of the nerve block defined as time from block performance (removal of the needle) until the indentation of the skin with a needle again feels sharp.
- **Maximum pain during tonic heat stimulation:** During this test, the participants' skin is heated to 45 °C for 30 seconds. The participants rates the pain elicited on a Visual Analogue Scale (VAS) using a ruler indicating ”no pain” = 0 mm at one end, and ”worst perceivable pain” = 100 mm at the opposite end. The duration of the nerve block measured by pain during tonic heat stimulation is defined as time from block performance (removal of the needle), until the tonic heat stimulation again elicits a painful response i.e. VAS >0.
- **Determination of warmth threshold (WDT)** represents the lowest temperature perceived as warm. Start temperature for the thermode is 32 ° C. The temperature rises by 1 ° C/sec during the threshold determination and 5 ° C/sec at the return to the starting temperature after the test. The participant is asked to press a button when the thermode feels warm while also finishing heat stimulation. If the limit of temperature increase beyond 52 ° C before recording a threshold value the thermode automatically returns to its starting temperature and 52 ° C is recorded. Each threshold value is calculated as the average of four stimulations; stimulations are presented 6-10 seconds apart. The duration of sensory block assessed by WDT, defined as the time from block performance until WDT is normalized.
- **Determination of heat-pain threshold (HPDT)** represents the lowest temperature perceived as painful. Start temperature for the thermode is 32 ° C. The temperature rises by 1 ° C/sec during the threshold determination and 5 ° C/sec at the return to the starting temperature after the test. The participant is asked to press a button, when the thermode feels painful while also finishing heat stimulation. If the limit of temperature increase beyond 52 ° C before recording a threshold value the thermode automatically returns to its starting temperature and 52 ° C is recorded. Each threshold value is calculated as the average of four stimulations; stimulations are presented 6-10 seconds apart. The duration of sensory block assessed by HPDT, defined as the time from block performance until HPDT is normalized.

The trial lasts from the introduction until the nerve block wears off (typically 10-14 hours). The participants will receive the above-mentioned treatments according to a computer-generated randomization list.

MEDICINE AND MEDICINE HANDLING

Trial medication

Active drug: **Ropivacaine 5.0 mg/ml.**
Dispensed as a clear colorless fluid for perineural or epidural use. Content: ropivacainehydrochlorid. Furthermore sodiumchloride, HCL, sodiumhydroxide and water.
Drug import to Denmark: Fresenius Kabi branch of Fresenius Kabi AB, Islands Brygge 57, 2300 Copenhagen S
Owner of the marketing rights: Fresenius Kabi AB. Marketing approval number: 45008.

Active drug: **Clonidine 150 µg/ml**
Dispensed as a clear colorless fluid for systemic use. Contains: clonidinhydrochlorid. Furthermore HCL (3.6%) (for pH neutralization).
Water for injection.

Drug import to Denmark: Specifik Pharma

Manufacturer: Boehringer Ingelheim Espana, S.A.

Owner of the marketing rights Germany: 6191514.00.01

Placebo: sodiumchloride 9 mg/ml. Inflution chloride. 1 l contains 9 g sodium chloride in sterile water. Electrolytes/l: 154 mmol chlorid og 154 mmol natrium. Isotonic. Osmolarity approximately 308 mmol/l.

Manufacturer: Skanderborg pharmacy

Blinding procedure, packaging and labeling

The trial is conducted in a blinded, randomized fashion. The randomization is done by a computer generated randomization list, generated by the Skanderborg pharmacy, who according to the randomization manufactures one box for each participant. The trial medicine will be packed and labelled by Skanderborg Pharmacy according to the applicable rules. The individual participant receives the trial medication as stated under **”General treatment plan and medical dosing”**.

All participants will receive an ABC with 20 ml of ropivacaine 5 mg/ml in each leg. As this treatment is the same in each leg and accordingly is not blinded, it will be taken from the department's own medical inventory.

Clonidine 150 µg/ml and isotonic saline are both clear, colorless fluids of identical appearance. Clonidine is produced by Boehringer Ingelheim and delivered in clear ampoules marked with a yellow and green line. The same identical empty ampoules are obtained from the manufacturer, and the pharmacy fills them with 1 ml isotonic saline. As the ampoules and content are identical in appearance, clonidine and placebo are blinded and relabeled by Skanderborg pharmacy, no distinction between the two substances are possible. Each box is packed with 2 x 1.0 mL ampoules, one of which is labeled "RIGHT LEG" and the other labeled "LEFT LEG” and randomization number, containing either clonidine 150 ug ml or placebo, according to randomization. On the trial day, the investigator notes the batch number and expiry, after which the medication is drawn into neutral syringes, labeled RIGHT LEG and LEFT LEG.

Two sets of sealed opaque envelopes are produced, containing information of which treatment the individual participants have received. The sponsor stores one set; the other set is stored by the principal investigator in a locked, safe place at the department of Anesthesiology, Zealand University Hospital, Køge, Denmark.

Other treatment

Other treatment deemed necessary for the participants, will be administered at the discretion of the investigator. The investigator is responsible of all medical decisions related to the trial. All medical treatment will be recorded in the Case Report Form.

Procedures in case of emergency

The Investigator ensures that procedures and expertise exists to manage any emergency that may arise during the trial.
The sealed opaque envelopes containing the assigned treatment can only be opened, if treatment of the participant depends on knowledge of the assigned treatment.
Immediate unmasking can be done without restrictions if deemed necessary without previous contact to monitor or sponsor. If the sealed opaque envelope is broken, date and reason for unmasking is registered in the CRF, and the envelope is signed by the investigator.

Medicine handling

All participant will receive an ACB with 20 ml of ropivacaine 5 mg/ml in each leg. As this treatment is the same in each leg and accordingly is not blinded nor randomized, it will be taken from the department's own medical inventory.
All medicine is handled by an investigator. Each box contains two ampoules; one ampoule containing clonidine and one ampoule of normal saline, for administration according to the labels "RIGHT LEG" or "LEFT LEG".
The investigator mixes the trial medication with 20 ml ropivacaine 0.5% and administers the medication immediately in the relevant legs according to the labels and randomization. Both drugs are clear colorless liquids and will appear identical in appearance and amount.

Medicine storage and tracking

Investigator ensures that the trial medication is kept in a safe place and only administered to participants in this trial. Only the principal investigator will handle the trial medication and batchnumber as well as expiry date will be noted in the trial master file. Investigator accounts for missing medication, as well as any discrepancy between delivered and returned medication.

Side effects

Side effects/AEs=adverse events

Adverse events are defined as any unwanted event, sign or symptom occurring during the trial related to the administration of the trial medication, whether this adverse event is deemed to be related to the trial medication or not. All adverse events are noted in the par-

ticipants Case Report Form. Should an adverse event occur more than 36 hours after administration of the trial medication, and no apparent association or connection to the trial medication can be made, it is not considered an adverse event.
Beginning and end, severity and symptoms related to the assumed adverse event following administration of the trial medication is noted. The severity of the adverse event and the association to the trial medication is evaluated according to the following guidelines by the investigator.

Guidelines for adverse events and causal relations to treatment

1. Not related – No temporal relation, other causes more likely.
2. Possibly related – Less clear connection, other causes may also be possible.
3. Probably related – Clear temporal connection with clinical improvement after termination of trial medication, and not reasonably explained by the participants known clinical condition.
4. Related – Clear temporal connection with retesting or clinical assessment.

Participants experiencing adverse events will be monitored with relevant clinical assessments and lab tests according to the treating doctor’s decision. All adverse events will be followed until satisfactory restitution or stabilization.
A Serious Adverse Event = SAE is an event that leads to risk of death or handicap for the participant (or their offspring) including, but not limiting to, an event that result in:

- Death
- Life threatening circumstance. Participant was, assessed by the investigator, at apparent risk of death by the adverse event as it occurred.
- Hospital admission
- Permanent invalidation
- Innate anomaly

A product resume for clonidine chapter 4.8 and the product resume for Ropivacaine chapter 4.8 from the department of health homepage as a reference document, when assessing whether a serious adverse event is expected or unsuspected.

Reduced sensitivity and motor deficits are a known effect of nerve blocks and will not be reported to the authorities unless strength and sensitivity is not regained within 36 hours after nerve block. There is a known risk of falling when nerve blocks involves n. femoralis, accordingly falling is not seen as an adverse event unless injury occurs in relation to the fall. The risk of falling is probably lower in relation to adductor channel blocks, all possible precautions will be taken to avoid falls.
Bradycardia, hypotension, sedation and dizziness are also known side effects of clonidine and will not be considered an adverse event.

Grading adverse events:
The investigator is obliged to investigate all clinical and objective reactions among the participants in the trial, and determine any causal relation to the trial medication. Reactions, if any, are graded according to the following scale:
1 = light
2 = moderate

3 = severe
4 = life threatening

Reporting AEs and SAEs

Investigator is responsible for registration of any AEs and SAEs in the participants Case Report Form.
Sponsor is responsible for ongoing monitoring of the trials risk/benefit relations. If events occur that may jeopardize the safety of the participants or the trial, the competent authority should **always** be informed. Similar reporting should also be done to all investigators and ethical committees.

In addition, the following guidelines of reporting to authorities are followed. All events and side effects of the medication are to be reported.

Sponsor is ongoing informed by the investigator on adverse events. By the completion of the trial, the final report should include a description of all adverse events.

Serious Adverse Events – SAE are reported to the sponsor by the investigator immediately. SAE´s are reported yearly to the ethics committee during the entire trial period along with a report on the safety of the participants.

Serious adverse reaction – SAR are reported by the sponsor (sponsor-investigator) yearly to the competent authority. The report also entails details on the safety of the participants.

Suspected unexpected serious adverse reaction – SUSAR are reported by the sponsor (sponsor-investigator**)** to the competent authority and the ethical committee within 24 hours. Report to the competent authority will be done using the electronic form for reporting SUSAR.

Deadly or life-threatening SUSAR´s are reported within **7 days** after sponsor's awareness of the event. Within **8 days** of the report, the sponsor must inform the competent authority of all relevant information on the follow-up of the event.

All other unexpected or serious suspected events must be reported to the authorities within 15 days of the event.

Any report to the authorities must be accompanied by a statement on the consequences for the trial.
It is recommended that sponsor (sponsor-investigator) informs the manufacturer of the event.

STATISTICAL ANALYSIS

Sample size calculation

A few studies have compared the duration of perineural clonidine with systemic clonidine, but these studies have examined combination with bupivacaine(4, 21), mepivacaine(3) and levobupivacaine(22) and not perineural ropivacaine. El Saied (9) investigated ropivacaine with clonidine as an adjuvant perineurally vs. ropivacaine alone in a study with 50 patients. The addition of clonidine as an adjuvant prolonged the sensory blockade 489-628 minutes (95% CI 90-187). In a yet unpublished study by our group on healthy volunteers, we found a sensory duration of an ACB with 20ml ropivacaine 5 mg/ml of 22 (SD 4) hours. Accordingly, we find that a 240 minutes prolongation of block duration will be clinically relevant. With a type 1 error risk of 5% and a type 2 error risk of 10% and an SD of 240 minutes, a total of 18 subjects is needed in this paired trial to show a difference of 240 minutes. In order to compensate for possible dropouts, we plan to include 21 participants.

Data handling

The trial ends when 21 participants are included.
Every participant's assessability in the statistical analysis will be determined before the code is broken. We will primarily perform an intention to treat analysis. Excluded participants and missing data will be described and accounted for, and if relevant (for instance in case of a partial nerve block) a per protocol analysis will be done.

Data is stored and analyzed on a computer in an anonymized form. Local data regulations will be adhered to.
Physical data in form of CRF will be kept in a locked cabinet in a locked office. Data is pseudo anonymized as the collected data is registered with a participant id number in the CRF.
The electronic pseudo anonymized data will be kept on a secure site on a computer, which only the principal investigator has access to.

Data is stored for 5 years after the completion of the trial, after which all paper material is maculated and electronic data is completely anonymized. The code to the assigned treatment will also be erased.

Results will be described using means, standard deviation to the mean, median and interquartile range where appropriate. A 5 % significance level will be employed. Normally distributed, continuous data are analyzed using paired t-tests, Whereas Wilcoxon signed-rank test will be applied to data violating the normality assumption. Any change to the statistical plan will be accounted for before publication.

DATAREGISTRATION AND RULES FOR CONTROL OF TRIAL PROCEDURES

The trial is conducted according to the rules applying for clinical trials on humans adhering to the Good Clinical Practice guidelines.
Investigator and sub investigators at University Hospital Zealand, Køge are responsible for handling and storing data according to the rules that apply. Data belongs to the principal

investigator Jakob Hessel Andersen, Department of Anesthesiology, University Hospital Zealand, Køge and sponsor Ole Mathiesen, Department of Anesthesiology, University Hospital Zealand, Køge.

Case Report Forms

For every participant included in the trial, a Case Report Form (CRF) will be used. The CRF will be signed by the investigator to confirm the authenticity of the data. Any Correction of data will be done by crossing out the wrong data in such a way, that the wrong data is still visible, and the correct data is written besides the crossed out wrong data. Corrections are signed and dated by the investigator or the sub investigator.
Source data are defined as the CRF.

Education

Investigator assures that the involved personnel is appropriately trained, instructed and has the necessary knowledge to conduct the trial.

FURTHER DEMANDS AND INFORMATION

Insurance

In the case of injury or harm caused directly or indirectly by the trial medication to the participants in this clinical trial, coverage on behalf of the investigator and sub investigators by the department of Anesthesiology, University Hospital Zealand, Køge is made, provided that the instructions given in this protocol and any amendments have been followed, and the trial have been conducted in concordance with applicable rules and accepted techniques. In the case of death or injury unrelated to the conduction of the trial, the participants are covered by University Hospital Zealand's insurance.

Publication plan
The investigator produces a report based on the data. This report will be send to the relevant authorities. The trial data and interpretation will also form the basis for a manuscript for publication with the following order of authors:

1. **Jakob Hessel Andersen**
2. **Pia Jæger**
3. **Tobias Laier Sonne**
4. **Ole Mathiesen**
5. **Jørgen B. Dahl**
6. **Ulrik Grevstad**

Trial results, both negative and positive, as well as inconclusive results will in any case be published.

1. Cuvillon P, Ripart J, Lalourcey L, Veyrat E, L'Hermite J, Boisson C, et al. The continuous femoral nerve block catheter for postoperative analgesia: bacterial colonization, infectious rate and adverse effects. Anesthesia and analgesia. 2001;93(4):1045-9.
2. Liu SS, Strodtbeck WM, Richman JM, Wu CL. A comparison of regional versus general anesthesia for ambulatory anesthesia: a meta-analysis of randomized controlled trials. Anesthesia and analgesia. 2005;101(6):1634-42.
3. Singelyn FJ, Dangoisse M, Bartholomee S, Gouverneur JM. Adding clonidine to mepivacaine prolongs the duration of anesthesia and analgesia after axillary brachial plexus block. Regional anesthesia. 1992;17(3):148-50.
4. Barioni MF, Lauretti GR, Lauretti-Fo A, Pereira NL. Clonidine as coadjuvant in eye surgery: comparison of peribulbar versus oral administration. Journal of clinical anesthesia. 2002;14(2):140-5.
5. Iskandar H, Benard A, Ruel-Raymond J, Cochard G, Manaud B. The analgesic effect of interscalene block using clonidine as an analgesic for shoulder arthroscopy. Anesthesia and analgesia. 2003;96(1):260-2, table of contents.
6. Eledjam JJ, Deschodt J, Viel EJ, Lubrano JF, Charavel P, d'Athis F, et al. Brachial plexus block with bupivacaine: effects of added alpha-adrenergic agonists: comparison between clonidine and epinephrine. Canadian journal of anaesthesia = Journal canadien d'anesthesie. 1991;38(7):870-5.
7. Singelyn FJ, Gouverneur JM, Robert A. A minimum dose of clonidine added to mepivacaine prolongs the duration of anesthesia and analgesia after axillary brachial plexus block. Anesthesia and analgesia. 1996;83(5):1046-50.
8. Bernard JM, Macaire P. Dose-range effects of clonidine added to lidocaine for brachial plexus block. Anesthesiology. 1997;87(2):277-84.

9. El Saied AH, Steyn MP, Ansermino JM. Clonidine prolongs the effect of ropivacaine for axillary brachial plexus blockade. Canadian journal of anaesthesia = Journal canadien d'anesthesie. 2000;47(10):962-7.
10. Erlacher W, Schuschnig C, Koinig H, Marhofer P, Melischek M, Mayer N, et al. Clonidine as adjuvant for mepivacaine, ropivacaine and bupivacaine in axillary, perivascular brachial plexus block. Canadian journal of anaesthesia = Journal canadien d'anesthesie. 2001;48(6):522-5.
11. Iskandar H, Guillaume E, Dixmerias F, Binje B, Rakotondriamihary S, Thiebaut R, et al. The enhancement of sensory blockade by clonidine selectively added to mepivacaine after midhumeral block. Anesthesia and analgesia. 2001;93(3):771-5.
12. Adnan T, Elif AA, Ayse K, Gulnaz A. Clonidine as an adjuvant for lidocaine in axillary brachial plexus block in patients with chronic renal failure. Acta anaesthesiologica Scandinavica. 2005;49(4):563-8.
13. Iohom G, Machmachi A, Diarra DP, Khatouf M, Boileau S, Dap F, et al. The effects of clonidine added to mepivacaine for paronychia surgery under axillary brachial plexus block. Anesthesia and analgesia. 2005;100(4):1179-83.
14. Reinhart DJ, Wang W, Stagg KS, Walker KG, Bailey PL, Walker EB, et al. Postoperative analgesia after peripheral nerve block for podiatric surgery: clinical efficacy and chemical stability of lidocaine alone versus lidocaine plus clonidine. Anesthesia and analgesia. 1996;83(4):760-5.
15. Casati A, Magistris L, Fanelli G, Beccaria P, Cappelleri G, Aldegheri G, et al. Small-dose clonidine prolongs postoperative analgesia after sciatic-femoral nerve block with 0.75% ropivacaine for foot surgery. Anesthesia and analgesia. 2000;91(2):388-92.
16. Madan R, Bharti N, Shende D, Khokhar SK, Kaul HL. A dose response study of clonidine with local anesthetic mixture for peribulbar block: a comparison of three doses. Anesthesia and analgesia. 2001;93(6):1593-7, table of contents.

17. Bharti N, Madan R, Kaul HL, Khokhar SK, Mishra S. Effect of addition of clonidine to local anaesthetic mixture for peribulbar block. Anaesthesia and intensive care. 2002;30(4):438-41.
18. Naja ZM, Ziade FM, El-Rajab MA, Naccash N, Ayoubi JM. Guided paravertebral blocks with versus without clonidine for women undergoing breast surgery: a prospective double-blinded randomized study. Anesthesia and analgesia. 2013;117(1):252-8.
19. Yazbeck-Karam VG, Siddik-Sayyid SM, Abi Nader EL, Barakat DE, Karam HS, Cherfane GM, et al. Supplementation of retrobulbar block with clonidine in vitreoretinal surgery: effect on postoperative pain. Journal of clinical anesthesia. 2011;23(5):393-7.
20. YaDeau JT, Paroli L, Fields KG, Kahn RL, LaSala VR, Jules-Elysee KM, et al. Addition of Dexamethasone and Buprenorphine to Bupivacaine Sciatic Nerve Block: A Randomized Controlled Trial. Regional anesthesia and pain medicine. 2015;40(4):321-9.
21. Culebras X, Van Gessel E, Hoffmeyer P, Gamulin Z. Clonidine combined with a long acting local anesthetic does not prolong postoperative analgesia after brachial plexus block but does induce hemodynamic changes. Anesthesia and analgesia. 2001;92(1):199-204.
22. Mannion S, Hayes I, Loughnane F, Murphy DB, Shorten GD. Intravenous but not perineural clonidine prolongs postoperative analgesia after psoas compartment block with 0.5% levobupivacaine for hip fracture surgery. Anesthesia and analgesia. 2005;100(3):873-8, table of contents.
23. Pratap JN, Shankar RK, Goroszeniuk T. Co-injection of clonidine prolongs the anesthetic effect of lidocaine skin infiltration by a peripheral action. Anesthesia and analgesia. 2007;104(4):982-3.
24. Jaeger P, Koscielniak-Nielsen ZJ, Hilsted KL, Grevstad U, Siersma V, Fabritius ML, et al. Effect of total dose of lidocaine on duration of adductor canal block, assessed by different test methods: A report of two, paired, blinded, randomized studies in healthy volunteers. [Submittet article under review]. In press 2016.

25. Jaeger P, Grevstad U, Henningsen MH, Gottschau B, Mathiesen O, Dahl JB. Effect of adductor-canal-blockade on established, severe post-operative pain after total knee arthroplasty: a randomised study. Acta anaesthesiologica Scandinavica. 2012;56(8):1013-9.
26. Jenstrup MT, JÆGer P, Lund J, Fomsgaard JS, Bache S, Mathiesen O, et al. Effects of Adductor-Canal-Blockade on pain and ambulation after total knee arthroplasty: a randomized study. Acta anaesthesiologica Scandinavica. 2012;56(3):357-64.
27. Jaeger P, Nielsen ZJ, Henningsen MH, Hilsted KL, Mathiesen O, Dahl JB. Adductor canal block versus femoral nerve block and quadriceps strength: a randomized, double-blind, placebo-controlled, crossover study in healthy volunteers. Anesthesiology. 2013;118(2):409-15.
28. Henningsen MH, Jaeger P, Hilsted KL, Dahl JB. Prevalence of saphenous nerve injury after adductor-canal-blockade in patients receiving total knee arthroplasty. Acta anaesthesiologica Scandinavica. 2013;57(1):112-7.
29. Williams BA, Hough KA, Tsui BY, Ibinson JW, Gold MS, Gebhart GF. Neurotoxicity of adjuvants used in perineural anesthesia and analgesia in comparison with ropivacaine. Regional anesthesia and pain medicine. 2011;36(3):225-30.
30. McCartney CJ, Duggan E, Apatu E. Should we add clonidine to local anesthetic for peripheral nerve blockade? A qualitative systematic review of the literature. Regional anesthesia and pain medicine. 2007;32(4):330-8.
