# Supplementary material for: Clonidine used as a perineural adjuvant to ropivacaine, does not prolong the duration of sensory block when controlling for systemic effects: A paired, blinded, randomized trial in healthy volunteers
Source: PLoS One. 2017 Sep 7;12(9):e0181351. doi: 10.1371/journal.pone.0181351 (PMC5589088; doi:10.1371/journal.pone.0181351)
Supplement: S1 Table — (PDF) [file pone.0181351.s005.pdf]

CONSORT 2010 checklist of information to include when reporting a randomised trial*

Section/Topic	Item No	Checklist item	Reported on page No
**Title and abstract**			
	1a	Identification as a randomised trial in the title	1
	1b	Structured summary of trial design, methods, results, and conclusions (for specific guidance see CONSORT for abstracts)	2
**Introduction**			
Background and objectives	2a	Scientific background and explanation of rationale	3-4
	2b	Specific objectives or hypotheses	4
**Methods**			
Trial design	3a	Description of trial design (such as parallel, factorial) including allocation ratio	5-7
	3b	Important changes to methods after trial commencement (such as eligibility criteria), with reasons	No change
Participants	4a	Eligibility criteria for participants	5
	4b	Settings and locations where the data were collected	5
Interventions	5	The interventions for each group with sufficient details to allow replication, including how and when they were actually administered	7-8
Outcomes	6a	Completely defined pre-specified primary and secondary outcome measures, including how and when they were assessed	8
	6b	Any changes to trial outcomes after the trial commenced, with reasons	No change
Sample size	7a	How sample size was determined	8
	7b	When applicable, explanation of any interim analyses and stopping guidelines	NA
Randomisation:			
Sequence generation	8a	Method used to generate the random allocation sequence	8
	8b	Type of randomisation; details of any restriction (such as blocking and block size)	8
Allocation concealment mechanism	9	Mechanism used to implement the random allocation sequence (such as sequentially numbered containers), describing any steps taken to conceal the sequence until interventions were assigned	8
Implementation	10	Who generated the random allocation sequence, who enrolled participants, and who assigned participants to interventions	5-8
Blinding	11a	If done, who was blinded after assignment to interventions (for example, participants, care providers, those	7-8

		assessing outcomes) and how	
	11b	If relevant, description of the similarity of interventions	8
Statistical methods	12a	Statistical methods used to compare groups for primary and secondary outcomes	8-9
	12b	Methods for additional analyses, such as subgroup analyses and adjusted analyses	NA
**Results**			
Participant flow (a diagram is strongly recommended)	13a	For each group, the numbers of participants who were randomly assigned, received intended treatment, and were analysed for the primary outcome	Fig 1
	13b	For each group, losses and exclusions after randomisation, together with reasons	Fig 1
Recruitment	14a	Dates defining the periods of recruitment and follow-up	10
	14b	Why the trial ended or was stopped	10
Baseline data	15	A table showing baseline demographic and clinical characteristics for each group	Table 1
Numbers analysed	16	For each group, number of participants (denominator) included in each analysis and whether the analysis was by original assigned groups	Fig 1
Outcomes and estimation	17a	For each primary and secondary outcome, results for each group, and the estimated effect size and its precision (such as 95% confidence interval)	Table 2
	17b	For binary outcomes, presentation of both absolute and relative effect sizes is recommended	NA
Ancillary analyses	18	Results of any other analyses performed, including subgroup analyses and adjusted analyses, distinguishing pre-specified from exploratory	NA
Harms	19	All important harms or unintended effects in each group (for specific guidance see CONSORT for harms)	12
**Discussion**			
Limitations	20	Trial limitations, addressing sources of potential bias, imprecision, and, if relevant, multiplicity of analyses	13-16
Generalisability	21	Generalisability (external validity, applicability) of the trial findings	13-16
Interpretation	22	Interpretation consistent with results, balancing benefits and harms, and considering other relevant evidence	13-16
**Other information**			
Registration	23	Registration number and name of trial registry	5
Protocol	24	Where the full trial protocol can be accessed, if available	1
Funding	25	Sources of funding and other support (such as supply of drugs), role of funders	Supporting material

*We strongly recommend reading this statement in conjunction with the CONSORT 2010 Explanation and Elaboration for important clarifications on all the items. If relevant, we also recommend reading CONSORT extensions for cluster randomised trials, non-inferiority and equivalence trials, non-pharmacological treatments, herbal interventions, and pragmatic trials. Additional extensions are forthcoming: for those and for up to date references relevant to this checklist, see www.consort-statement.org.
